# A large collection of bioinformatics question–query pairs over federated knowledge graphs: methodology and applications

**DOI:** 10.1093/gigascience/giaf045

**Published:** 2025-05-16

**Authors:** Jerven Bolleman, Vincent Emonet, Adrian Altenhoff, Amos Bairoch, Marie-Claude Blatter, Alan Bridge, Séverine Duvaud, Elisabeth Gasteiger, Dmitry Kuznetsov, Sébastien Moretti, Pierre-Andre Michel, Anne Morgat, Marco Pagni, Nicole Redaschi, Monique Zahn-Zabal, Tarcisio Mendes de Farias, Ana Claudia Sima

**Affiliations:** SIB Swiss Institute of Bioinformatics, 1015 Lausanne, Switzerland; SIB Swiss Institute of Bioinformatics, 1015 Lausanne, Switzerland; SIB Swiss Institute of Bioinformatics, 1015 Lausanne, Switzerland; SIB Swiss Institute of Bioinformatics, 1015 Lausanne, Switzerland; SIB Swiss Institute of Bioinformatics, 1015 Lausanne, Switzerland; SIB Swiss Institute of Bioinformatics, 1015 Lausanne, Switzerland; SIB Swiss Institute of Bioinformatics, 1015 Lausanne, Switzerland; SIB Swiss Institute of Bioinformatics, 1015 Lausanne, Switzerland; SIB Swiss Institute of Bioinformatics, 1015 Lausanne, Switzerland; SIB Swiss Institute of Bioinformatics, 1015 Lausanne, Switzerland; SIB Swiss Institute of Bioinformatics, 1015 Lausanne, Switzerland; SIB Swiss Institute of Bioinformatics, 1015 Lausanne, Switzerland; SIB Swiss Institute of Bioinformatics, 1015 Lausanne, Switzerland; SIB Swiss Institute of Bioinformatics, 1015 Lausanne, Switzerland; SIB Swiss Institute of Bioinformatics, 1015 Lausanne, Switzerland; SIB Swiss Institute of Bioinformatics, 1015 Lausanne, Switzerland; SIB Swiss Institute of Bioinformatics, 1015 Lausanne, Switzerland

**Keywords:** knowledge graphs, Resource Description Framework (RDF), federated SPARQL, query editor, metadata

## Abstract

**Background:**

In recent decades, several life science resources have structured data using the same framework and made these accessible using the same query language to facilitate interoperability. Knowledge graphs have seen increased adoption in bioinformatics due to their advantages for representing data in a generic graph format. For example, yummydata.org catalogs more than 60 knowledge graphs accessible through SPARQL, a technical query language. Although SPARQL allows powerful, expressive queries, even across physically distributed knowledge graphs, formulating such queries is a challenge for most users. Therefore, to guide users in retrieving the relevant data, many of these resources provide representative examples. These examples can also be an important source of information for machine learning (for example, machine-learning algorithms for translating natural language questions to SPARQL), if a sufficiently large number of examples are provided and published in a common, machine-readable, and standardized format across different resources.

**Findings:**

We introduce a large collection of human-written natural language questions and their corresponding SPARQL queries over federated bioinformatics knowledge graphs (KGs) collected for several years across different research groups at the SIB Swiss Institute of Bioinformatics. The collection comprises more than 1,000 example questions and queries, including almost 100 federated queries. We propose a methodology to uniformly represent the examples with minimal metadata, based on existing standards. Furthermore, we introduce an extensive set of open-source applications, including query graph visualizations and smart query editors, easily reusable by KG maintainers who adopt the proposed methodology.

**Conclusions:**

We encourage the community to adopt and extend the proposed methodology, towards richer KG metadata and improved Semantic Web services.

**URL:**  https://github.com/sib-swiss/sparql-examples.

## Introduction

The accuracy and real-world applicability of question-answering systems over structured data crucially depend on the availability of high-quality, representative question–query pairs over diverse datasets. Resources, such as UniProt [1], have been used in bioinformatics for many years, where they facilitate data integration and exploration through (potentially federated) SPARQL queries. However, a long-standing challenge is making this wealth of data usable by a wider range of users, including researchers without technical training. Writing SPARQL queries is outside the competence of most life scientists because it requires both knowledge of the query language itself and understanding of the knowledge graph (KG) schema, for example, a Resource Description Framework (RDF) graph data model. Many solutions have thus been investigated, including natural language interfaces (NLIs) to KGs. The recent progress in large language models (LLMs) has boosted performance across a wide range of natural language processing tasks, including promising results in LLM-based knowledge graph question answering (KGQA) systems [2]. A recent study highlights that unified models, trained across diverse datasets stemming from distinct KGs, eliminate the need for separate models for each KG and can therefore significantly reduce the overall costs of fine-tuning and deploying such models [3]. Therefore, a key requirement for the development of novel, LLM-based KGQA systems is the availability of high-quality, representative public datasets of questions and corresponding SPARQL queries across a wealth of diverse KGs.

Capturing user intent and translating questions becomes significantly more complex when considering multiple, distributed scientific KGs such as those comprising the SIB Swiss Institute of Bioinformatics Semantic Web of Data. Although federated SPARQL queries are very powerful because they enable joint querying of physically distributed KGs, the challenge lies in formulating such queries. Federated queries are inherently complex because they require users not only to understand the data models of the individual sources involved in the query, but also to be aware of how these sources interconnect (i.e., how they can be joined). Hence, the availability of examples to guide users—or of a system supporting the automatic translation of user questions to federated queries—is critical. However, federated queries have been mostly neglected so far in existing collections of natural language questions and equivalent SPARQL queries.

The SIB hosts a number of high-quality, curated, and interoperable life science KGs [4] that are accessible via public SPARQL endpoints, i.e., web addresses that can receive and process SPARQL queries. Each of these KGs is independently managed by different research groups within the institute. However, a common point across all resources is that they each maintain a set of representative questions and their equivalent SPARQL queries, collected over time. These are made available to inspire and help users formulate new queries over the respective KG data. Importantly, the resources also include examples of federated queries, which make it possible to join information from their KG with those of other, complementary KGs, from the SIB catalog and beyond (e.g., Wikidata [5], IDSM [6], ChEMBL [7]). Prior to the work presented in this paper, the examples were provided in heterogeneous and sometimes non-machine-readable formats (e.g., as plain text in a static webpage). However, to facilitate interoperability and more generally the reusability of all examples as a large, uniform collection, it is of paramount importance to standardize how queries are represented, in agreement with the FAIR data principles (findable, accessible, interoperable, and reusable), allowing their automatic discoverability by both users and services.

In this paper, we introduce the growing collection of over 1,000 human-written, real-world question–query pairs, including almost 100 federated queries (i.e., queries that ask questions requiring at least 2 sources jointly), collected over several years across 11 interoperable KGs. Additionally, we describe the methodology we have applied across SIB to represent, store, and test example queries by adopting different (meta)data standards. Importantly, we show that this standardization makes it easy to find examples across different endpoints, and to set up, run, and maintain services that use these examples. These services include powerful user-facing applications, such as search engines or automatically generated web visualizations. The rapid development and uptake of these services across different SIB resources directly prove the value of adopting FAIR data principles and metadata standardization. To our knowledge, ours is the most comprehensive portfolio of bioinformatics examples and services facilitated by FAIR KG (meta)data across distributed KGs. Both the collection of example questions–SPARQL queries, as well as all the developed services leveraging these examples, are available open source and can be adapted to other KGs by following our recommended metadata format.

We encourage the community to engage with the collection of examples, adopt and further extend our proposed format for describing SPARQL examples, and finally reuse and improve the services presented in this work. The SIB community will continue updating the catalog with new examples. Efforts towards standardization will not only contribute towards FAIRer KGs, but will also help build a solid foundation for a new generation of Semantic Web technologies, such as LLM-based natural language interfaces over KGs.

## Sources

At the time of writing, we have collected contributions from 10 large-scale KGs across different SIB groups. Additionally, we integrated contributions from one external KG, “dbgi,” with examples contributed by external collaborators from the Digital Botanical Gardens Initiative [8]. The collection is continuously updated with new examples. An up-to-date overview of the number of queries, including the number of federated queries, is generated automatically from the collection and can be visualized live online [9].

For details on the SPARQL endpoints maintained by the SIB, we refer the reader to the SIB Semantic Web of Data [10]. Among the contributing KGs, we can mention UniProt—currently the largest publicly available KG—with over 168B triples and a total of 61 contributed example queries. To our knowledge, this collection is the most comprehensive collection of real-world SPARQL queries examples to date in bioinformatics.

**Figure 1:**
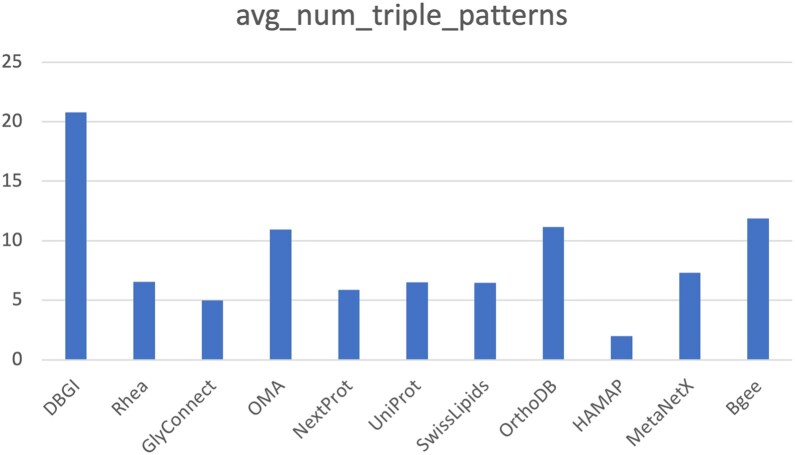
Average number of triple patterns per query in each of the KGs contributing to the examples collection.

As an estimate of the complexity of the queries in this collection, we show the average number of triple patterns (TP) per query for each endpoint, included in the collection in Fig. 1. The number of TPs is a good approximation for the complexity of a query, because it indicates the number of hops (joins) required to compute an answer over the graph. Most queries have around 6 TPs on average, with the notable exception of the DBGI, for which queries have a much higher complexity, due to the complexity of the data model, but also due to federation with Wikidata, which is present in most examples. On the other end, HAMAP includes only a few very simple examples because it has a relatively small schema (encompassing only 7 classes) for describing annotation rules. We note that most existing large-scale collections focus on simple queries, i.e., queries with at most 3 TPs (for examples, see Related Work). Additional information on query complexity (for example, total number of aggregations, such as SUM, COUNT, etc.) is generated automatically from the catalog and available online [9].

## Methodology

Standardization is at the core of our approach. To structure and describe the question–query pairs, we rely on the SHapes Constraint Language (SHACL) vocabulary, which is RDF based [11]. In addition to SHACL and RDF Schema (RDFS) [12], we describe and annotate the examples with Schema.org. Standardizing how we represent the examples is crucial because they can target different and independent data sources managed by distinct groups and organizations. A standardized, machine-readable representation enables both users and services to discover and smoothly use the question–query descriptions that are defined in RDF and stored in distributed data stores accessible with SPARQL. More precisely, example question–query pairs can be stored with their associated metadata in the same RDF store, consequently making them accessible via the same SPARQL endpoint. As a result, this standardization and infrastructure facilitate the automatic testing of queries, as well as the deployment of services with minimal code modifications across endpoints, such as the generation of user-friendly Web pages with query descriptions and graphical visualizations (see Applications).

**Figure 2:**
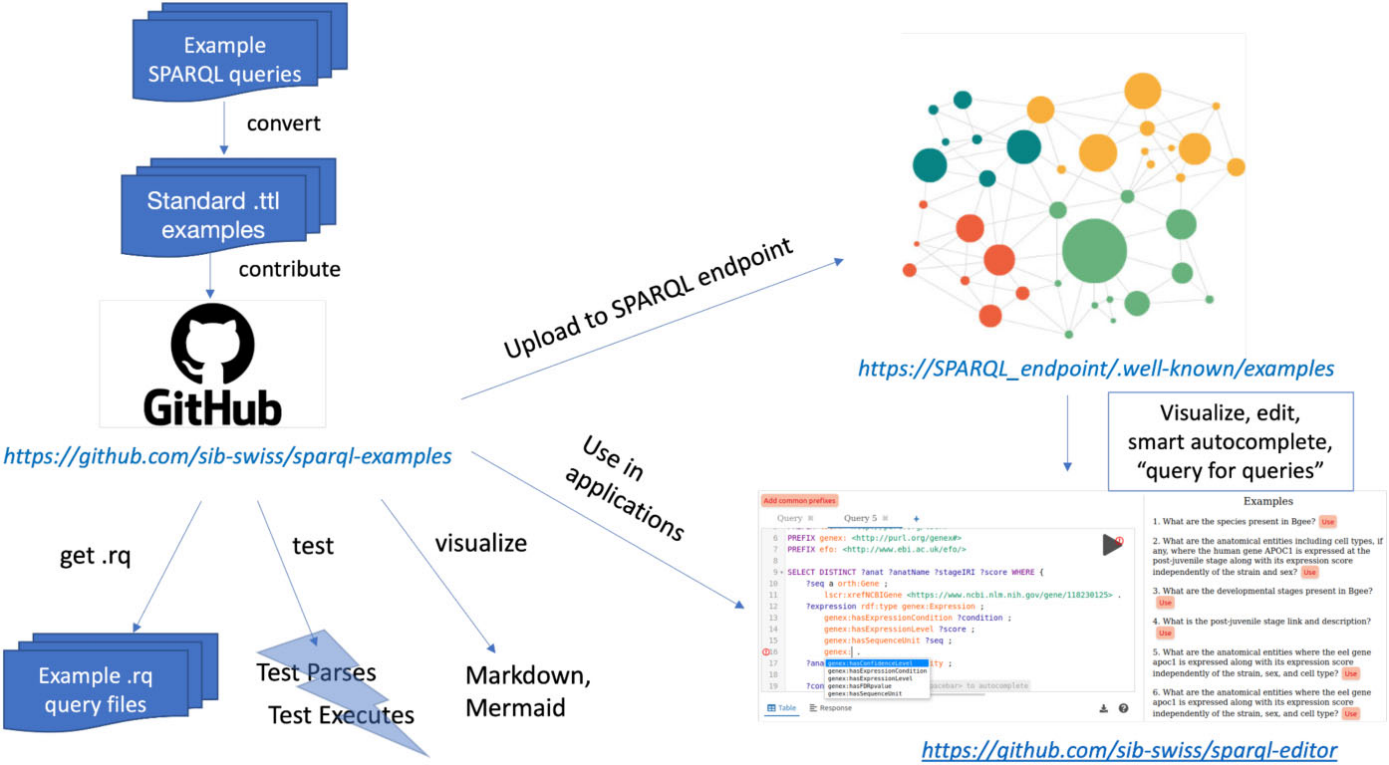
End-to-end workflow, from example contributions to services that uniformly consume examples across distinct SPARQL endpoint (e.g., sparql-editor and Bio-Query template search).

Figure 2 illustrates the end-to-end workflow, starting from examples contributed by KG maintainers to services consuming the examples from the named graph they are published in.

### Implementation

We collaborate in an open GitHub repository [13]. The repository is the “single source of truth,” where all SPARQL examples are collected via contributions from data store maintainers. Each example is stored in a separate file using the Terse RDF Triple Language (Turtle, i.e., “.ttl”) syntax and file format [14], within a folder named according to the resource that contributed the example. For example, all Bgee queries can be found in [15].

Each Turtle file stores a single example, which is represented by:

- The type of the query (e.g., Ask, Select, Describe).
- The description of the query (e.g., the question in plain text), including a language tag.
- The SPARQL query itself.
- The target SPARQL endpoint(s) where the query can be executed.
- Additional keywords (tags).

Each question–query pair is defined as a SPARQLExecutable SHACL concept [11]. Note that although SHACL is documented as a technical report and a W3C recommendation in [16], the vocabulary terms we use are only fully described in its RDF serialization, either Turtle or RDF/XML, in [17]. We also specify the SPARQLExecutable subtype, such as SPARQLAskExecutable and SPARQLSelectExecutable, i.e., executable queries based on an ASK and SELECT SPARQL query, respectively. SHACL as standard is aimed at describing shapes that data may conform to. These shapes may then be used for validation, user interfaces, and optimizations. We use the SHACL standard because each SPARQL query can be seen as a declaration of a data shape that a SPARQL engine instantiates from the data stored within it. Furthermore, all of the fields (b)–(e) above are represented in a structured, standardized, and machine-readable format: the plain English question or description of the query can be described via an rdfs:comment, the query itself via sh:select, sh:ask, sh:update, sh:construct, or sparql-examples:describe [18] (according to the type of the SPARQL query, i.e., ASK, SELECT, etc.). For multilingual support in the examples, all questions are annotated with a corresponding language tag (e.g., “@en” for English). The set of queries in this work only includes ASK, SELECT, and DESCRIBE queries. As DESCRIBE queries are defined in SPARQL 1.1 and cannot be used in SHACL, we introduce a new property and concept to represent this type of query, i.e., sparql-examples:describe where the sparql-examples: prefix corresponds to https://purl.expasy.org/sparql-examples/ontology#. The target SPARQL endpoint(s) are described with schema:target. Note that there can be several such endpoints where the query is directly executable. The query reusability (without any modifications) stems from the adoption of the FAIR principles in the design of KGs across the entire SIB Semantic Web of Data, which makes them uniformly queryable when requesting the same type of information. For example, the query defined at [19] (see also the SPARQL query shown in Listing 1) is usable as is in at least three SPARQL endpoints, namely Bgee, OMA, and UniProt. In the case of this example, it retrieves the list of species in each KG. The schema:target shows where users can run these queries and also demonstrates the benefit of sharing knowledge representation of common concepts and values. Finally, example queries can optionally be annotated with additional keywords (tags) using the schema:keywords property.

To further illustrate how question–query pairs are structured, Listing 1 shows another example of a SPARQL query that retrieves all taxa in UniProt and its corresponding question in plain English. The SPARQL query is defined using the sh:select relation, while the corresponding natural language question (i.e., the question in plain English that corresponds to the query) is always assigned to the rdfs:comment relation. In the case of a DESCRIBE query, the main change is replacing sh:select property with spex:describe, followed by the text of the query, i.e., “DESCRIBE <URL>.” The type of the DESCRIBE example query will be only sh:SPARQLExecutable. A concrete case can be seen at [20].

Listing 1: Question–query pair to select all UniProt taxa. We define this as a sh:SPARQLSelectExecutable where the question is assigned to rdfs:comment (with an English language tag annotation), while its corresponding SPARQL query is sh:select. The schema:target relation is used to define the target SPARQL endpoint where the query is executable. Optionally, the sh:prefixes relation is also stated to report all prefixes needed to run the query. This example has been uploaded to the UniProt SPARQL endpoint and can be viewed and executed at [19].

PREFIX ex: <https://sparql.uniprot.org/.well-known/sparql-examples/>

PREFIX sh: <http://www.w3.org/ns/shacl#>

PREFIX rdf: <http://www.w3.org/1999/02/22-rdf-syntax-ns#>

PREFIX rdfs:<http://www.w3.org/2000/01/rdf-schema#>

ex:001 a sh:SPARQLSelectExecutable, sh:SPARQLExecutable;

 sh:prefixes _:sparql_examples_prefixes;

 rdfs:comment “Select all taxa from the UniProt taxonomy”@en;

 sh:select “”“”PREFIX up: <http://purl.uniprot.org/core/>

SELECT ?taxon

FROM <http://sparql.uniprot.org/taxonomy>

WHERE

{

 ?taxon a up:Taxon.

}“”";

 schema:target <https://sparql.uniprot.org/sparql/;>

 schema:keywords “taxa.”

After describing the SPARQL examples, we also store them in a standardized location directly as a subgraph within the KG that they target—i.e., as part of the same SPARQL endpoint as the data they act on—so as to enhance their findability. This allows both users and services to uniformly retrieve examples of interest, or to aggregate information about the collection of examples available in each resource, by directly querying the SPARQL endpoints themselves. For example, users can query about all examples that target a specific endpoint, or all examples—across endpoints—that are annotated with a keyword of interest, by running a dedicated select SPARQL query, e.g., filtering on the schema:keywords property or on the text of the question itself (see Listing 2). Our GitHub repository includes more such pointers in the section “Querying for queries.” In addition, the standardized representation enables reusability of services across KGs with minimal modifications and without reliance on a centralized repository. We also provide in our GitHub repository [13] a script to merge the examples targeting a given endpoint as a single .ttl file that can be uploaded to a dedicated subgraph (e.g., as a named graph) within each KG. Across SIB, we have chosen to upload examples to a named graph composed of the SPARQL endpoint web address and the “.well-known/sparql-examples” suffix. Examples can then be assigned a unique identifier, more precisely, an Internationalized Resource Identifier (IRI), which may also be resolvable, i.e., allow access to the query using standard HTTP(S) for resource maintainers that want to associate specific webpages to the queries. For example, the query that retrieves all taxa over the UniProt SPARQL endpoint can be accessed at [19].

Once uploaded in the SPARQL endpoint, the examples themselves can be processed (e.g., to search for relevant examples on a given topic) through simple SPARQL queries. Listing 2 shows how to retrieve example question–query pairs where the question contains the keyword “species” from a given endpoint. The following query can be directly executed, for example, over the UniProt SPARQL endpoint [21] or the Bgee SPARQL endpoint [22]:

Listing 2: Query to retrieve all examples that include questions containing the keyword “species” from a SPARQL endpoint where formatted SPARQL examples have been uploaded.

PREFIX rdfs: <http://www.w3.org/2000/01/rdf-schema#>

PREFIX sh: <http://www.w3.org/ns/shacl#>

SELECT * WHERE {

 ?ex sh:select ?query.

 ?ex rdfs:comment ?question.

 FILTER (contains(?question, “species”))}

Finally, the landing page of our open GitHub repository provides an extensive description of how to apply our methodology to other KGs (RDF data stores). We include instructions and automated scripts to help describe new examples (i.e., query–question pairs), as well as explaining where such examples should be stored, how they can be queried (i.e., “querying for queries”), tested automatically, etc. To help KG maintainers check whether their endpoint meets all criteria required for metadata annotation, we have also developed an automated checker available online [23]. The checker only requires a SPARQL endpoint URL to validate the existence of properly formatted metadata, such as SPARQL examples and the Vocabulary of Interlinked Datasets (VoID) [24] description, in the given endpoint. VoID is an RDF Schema vocabulary for expressing metadata about RDF datasets. It may be used to quantitatively describe relations between different datasets, or their subsets. For example, a VoID description will list classes and predicates used in a dataset and their cardinalities, i.e., how often they occur. For endpoints which do not have the full metadata available, the checker suggests concrete steps that can be taken, for example, to add the corresponding VoID description to the endpoint by using our open-source VoID generator [25].

## Applications

In this section, we provide several applications facilitated by the collection of example queries, such as:

- Automated query testing across SIB endpoints (helping maintain query examples).
- Automatically generating graph visualizations of queries.
- Uniform query search and editing with a reusable SPARQL editor.
- Searching for scientific questions in the Bio-Query template interface.

All the applications can be deployed across the entire collection of questions and queries due to the standardized representation of examples that we have designed. The applications can be reused over any question–query collections that follow the approach described in the Methodology section. All of the applications support KG maintainers in providing users with better support for querying their endpoints, addressing the challenges we have identified in the Introduction. More specifically, automated query testing ensures that the examples provided by maintainers remain up-to-date as the underlying data evolves. Query search and editing ensures that users with basic familiarity with SPARQL can find a relevant existing query and adapt it to their needs. Graph visualizations of queries further support users with little SPARQL knowledge to understand the general data flow of a query, while Bio-Query ultimately enables users to uniformly search for queries across the entire catalog and to adapt existing templated queries to their own information needs. We describe these applications in more detail in the following sections.

### Tooling to help maintain SPARQL query examples

To help teams maintain their own SPARQL query examples, we provide a set of tools in a separate GitHub repository [26]. This includes testing for continuous integration, visualizations to help understand the queries, and a set of automated fixes that can remove common errors. We provide details on each of these components in the following sections.

#### Automated testing

Each SPARQL query is tested for syntactical correctness using two different parsers: the first provided by the Apache Jena [27] project, and a second by the eclipse RDF4j [28] project. The metadata about the query is tested using a set of SHACL rules with the RDF4j implementation of SHACL. These rules check if the structure of the metadata around the query matches the expected format, avoiding errors in the contributed examples. For example, the presence of all mandatory fields (query ID, query type, comment, target) is checked. Furthermore, the rules check if the query matches the metadata—for example, that all queries declared to be federated queries actually contain a SERVICE clause in the query text.

Optionally, we can also execute all queries on their remote target endpoints. We chose to only check that the queries retrieve at least one result, by programmatically changing the queries. More specifically, we add a limit that restricts the queries to only retrieve one result (i.e., a LIMIT [29] clause with a value of 1). This avoids introducing non-essential load on the tested SPARQL servers because some queries would result in long-running times or high computational load. Finally, all SPARQL endpoints used in federated queries are tested to be alive and responsive to SPARQL queries. These tests are written in Java using JUnit 5 Jupiter [30] as the test framework. Tests that do not require remote SPARQL endpoints are deployed as GitHub actions that run on each push to the shared GitHub repository, ensuring the validity of newly contributed queries. Tests that require network or that access the remote SPARQL endpoints are only run on demand by the query writers and can be used as regression tests when changes are made to the target repositories (i.e., to ensure that existing queries are still valid). Many of the query examples are relatively complex and would therefore add significant traffic to the remote endpoints if executed frequently. We thus only run these tests on demand.

#### Enabling query visualization

**Figure 3:**
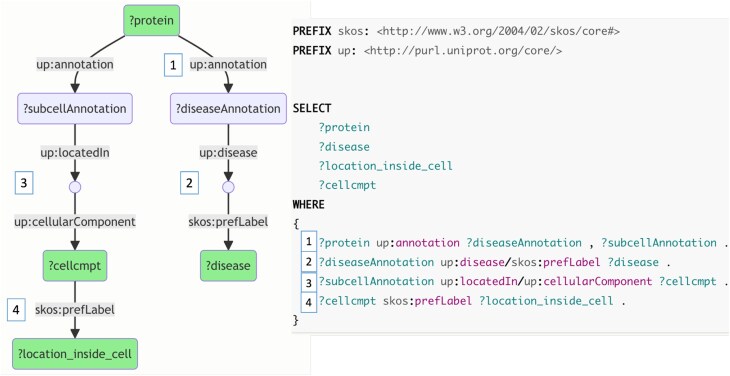
A graphical representation of [36] retrieving disease-related proteins that are known to be located within the cell. This visualization is available in the GitHub pages of the collection of SPARQL examples [37]. In the figure, green cells show the projected variables, i.e., the variables that are selected to be included in the final result. The small circles act as intermediary, undefined variables (i.e., intermediary blank nodes) introduced by decomposing property paths into individual triple patterns. The gray boxes shown as edge labels are the properties in the query triple patterns. In this figure, the numbered boxes shown on the left side of edges help to match the visualization to the corresponding triple patterns in the query shown on the right side.

Complex SPARQL queries are difficult to follow for the majority of users. To assist in understanding the existing question–query examples, we automatically generate Markdown [31] files with visual graphs and descriptions for each example query. To visualize these, we use Mermaid [32], a diagramming and charting tool inspired on the Markdown text syntax. Namely, we automatically generate for each example query a corresponding visual graph in Mermaid. The generated markdown files can be displayed as GitHub pages [33] or as web pages in general (i.e., rendered HTML [34]). These web pages can be then accessible, for example, via the web address used as their IRI. To facilitate the findability and understanding of the examples, we make the full collection, including Mermaid visualizations for each query, available online in GitHub pages [35]. Figure 3 shows a simple Mermaid-based graph corresponding to an example query that asks for disease-related proteins located inside the cell. This query can be executed at the UniProt SPARQL endpoint.

#### Fixing query examples to be fully compliant with SPARQL 1.1

A number of query engines have extended SPARQL 1.1 in non-standard ways. We implemented a module that attempts to “fix” these queries to be fully compliant with the SPARQL 1.1 specification. For example, Anzo and Blazegraph have implemented an extension for named subqueries. This named subquery extension is widely used in the Wikidata community. Nevertheless, these queries can often be semantically rewritten by following the SPARQL 1.1 specification. More precisely, our “fixer” approach parses the queries with the Blazegraph SPARQL parser and browses specific abstract syntax trees to look for such named subqueries and replace them with SPARQL 1.1 standard-compliant subqueries.

Another common mistake is that query examples might miss the prefix declaration required to make a query run. We store the common prefixes used in our resources, with an option for project-specific prefixes; we can easily add missing ones to a query without further user interaction.

### Query editing and searching

**Figure 4:**
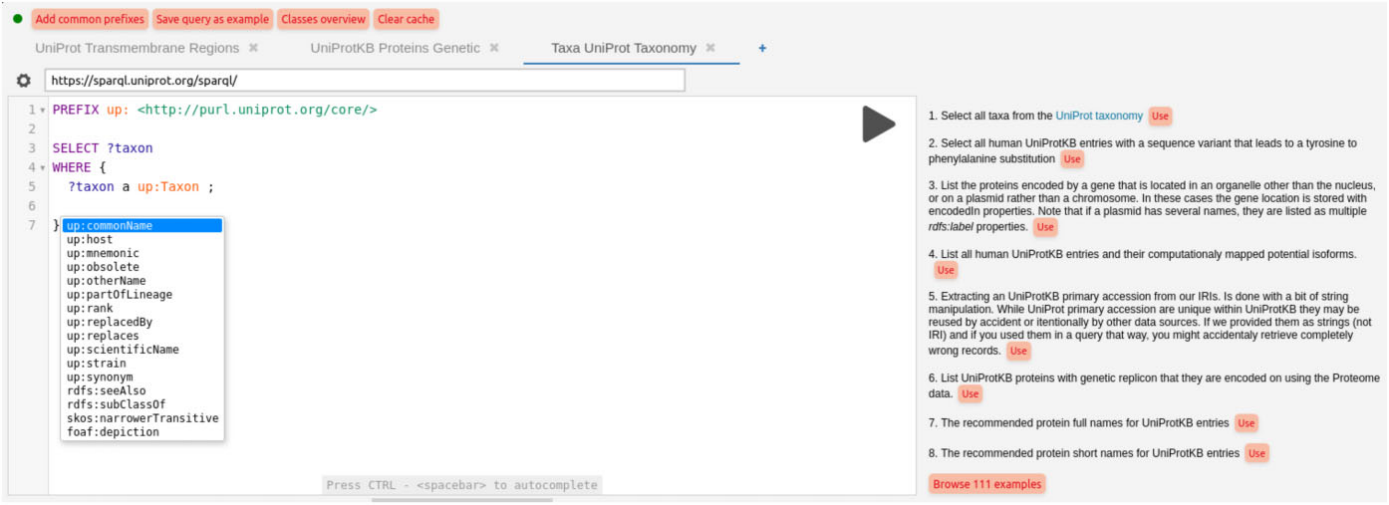
SPARQL query editor with context-aware autocomplete for the UniProt SPARQL endpoint. The list of query examples, classes, and properties is automatically retrieved from the endpoint.

We extend the widely used Yasgui SPARQL environment [38] with a new section showing the queries in use. The code for this extension is available as a package at the npm software registry [39] and can be easily deployed by SPARQL endpoint maintainers using a single custom HTML element <sparql-editor/>. We welcome contributions to develop this further at [40]. The editor, available online [23] and illustrated in Figure 4, includes several useful features. It automatically pulls query examples from any SPARQL endpoint of interest (configurable at the top of the editor page) and presents them to the user alongside the editor, so users can easily reuse these queries to write their own by editing or extending them. It also provides a precise autocomplete based on the VoID descriptions that summarize an endpoint content. Whenever available, these descriptions are retrieved by the editor that queries the endpoint itself. The main information of interest processed by the editor from the VoID descriptions are the classes and properties that connect them. The VoID description is directly extracted from the SPARQL endpoint that is used by our extended Yasgui environment, therefore accurately reflecting the actual data. This ensures that only properties that are indeed stated and applicable according to the schema and the known type of variable are suggested in the autocomplete dropdown menu. For further details on the VoID description generator used, see the corresponding GitHub repository [25].

In contrast, the authors of [41] propose a context-aware autocomplete approach that queries the SPARQL endpoint data directly. While effective for certain use cases, this method can face scalability challenges when dealing with large KGs not hosted in QLever triplestores. Our approach, which leverages precomputed metadata about the KG, ensures rapid responses across any triplestore, but requires endpoint maintainers to generate and maintain the VoID descriptions. These approaches are not mutually exclusive; we are exploring the integration of QLever's method into our SPARQL editor. This hybrid solution would enable the editor to dynamically select the most suitable autocomplete strategy for each endpoint, balancing scalability and responsiveness.

### Integration in Bio-Query

**Figure 5:**
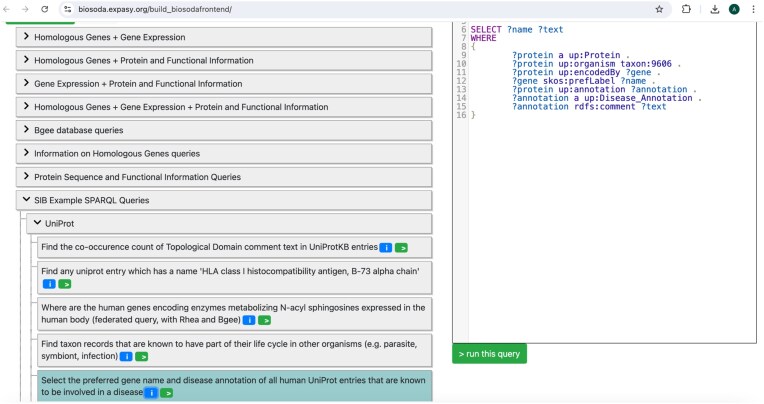
Example UniProt entry in the Bio-Query interface, integrated from the collection of SIB Example SPARQL queries. The collection is processed from the central GitHub repository in order to produce the JSON representation required by the interface. The standardization of the examples minimizes the effort to integrate these in the common interface, which can then act as a central hub to search for, and adapt, existing examples across SIB KGs.

The standardized representation of example queries facilitates their use in applications downstream. As an example, we have also incorporated the collection as is in the Bio-Query [42] interface available online [43]. Figure 5 shows an example question and query from the collection. The questions are automatically assigned to categories named according to the resource that contributed them. The collection can be found under the “SIB Example SPARQL queries” category in the public Bio-Query interface.

## Challenges and lessons learned

Efforts towards standardization are always faced with challenges at multiple levels. We have found that, even with community agreement on a common metadata format, simple details, such as non-uniform HTTP response headers expected across the different endpoints, can still hamper the uniform processing of the metadata. Moreover, even the queries themselves—although in principle portable across different triplestores—are not always reusable across different implementations. This is because, as mentioned previously, some query engines have extended SPARQL 1.1 in non-standard ways. An example is the way that “magic triples” are used to provide query execution hints [44] in Blazegraph or AWS Neptune. These “magic triples” need to be removed when moving to a different SPARQL endpoint because they would otherwise refer to triples that do not exist, and would therefore break the original queries (i.e., their execution would give empty results).

At a higher level, one of the biggest challenges we still face is that the collection of questions and queries was designed to provide generic guidelines for users, and not to be consumed by machines directly. This poses a set of unique challenges also in reusing them for the purpose of training and evaluating machine learning algorithms to automatically translate questions into equivalent SPARQL queries. End-user questions are often more abstract than their corresponding SPARQL queries, because the information searched by the user is not explicitly defined in the question. For example, let us consider the question: “What are the species available?” In this question we do not know what is the exact information the user is looking for. Is the user searching for common names or scientific names of species, or both? Is the user looking for a specific list of species identifiers of a given taxonomy? On the other hand, in a SPARQL query we often define the exact information to be retrieved (i.e., query projection). This impacts the retrieved results and potentially their amount. Therefore, while for an end-user the exact projected variables do not seem to be an essential point in the translation of a question to a query, for automatically evaluating an ML system, the set of projected variables must be precisely defined in the question (e.g., “return only the species scientific names,” “return only proteins and their associated gene names”) as opposed to being loosely defined, which would hamper a precise evaluation. Some questions in the catalog have also been designed in sequence, such that the text of one question is a follow-up to a previous one (e.g., “Same as previous but with P0ABU7 as a query”). These questions would need to be rephrased such that they are self-contained, given that most existing ML systems for translating natural language questions to queries treat each question as independent of the others.

Finally, an added challenge (common to all collections of question-query pairs) is that the SPARQL endpoints the queries target might change or even be no longer available, rendering the queries harder to execute or reproduce. We acknowledge that ensuring long-term preservation of the corresponding data is a difficult task; however, precisely due to this reason we cannot maintain dedicated copies of the datasets targeted by the collection of queries. Instead, we rely on the resource providers to provide a long-term solution, which can be, for example, automatically redirect the existing SPARQL endpoint link to a site where the data are available at least in an archived format, from where users can deploy it locally.

Nevertheless, in spite of these challenges, the portfolio of reusable services that we have developed and deployed across independently managed SIB endpoints attests to the fact that even small steps towards standardization can have an important impact.

## Related work

Many existing works have proposed collections of natural language questions and corresponding SPARQL queries, usually designed as benchmarks to facilitate the task of automatically translating questions to queries or to evaluate federated query optimizers. However, existing collections suffer from shortcomings, which we mention below:

1. Non-federated. To the best of our knowledge, existing peer-reviewed published benchmarks focusing on translating questions to queries do not consider federated queries and are usually centered around a single endpoint. We can mention here the examples of [45, 46]. Even multi-dataset collections usually effectively address one single endpoint at a time, such as [45–47]. Consequently, existing KGQA systems also focus on simple, non-federated queries [48], often solely over DBPedia or Wikidata [49]. The existing collections of federated SPARQL queries, such as [50, 51], are designed to merely evaluate SPARQL federation engines and do not include corresponding natural language questions.
2. Non-representative. Crafting a large-scale benchmark manually is an extremely costly endeavor; therefore, large collections (see [46]) have been generated semi-automatically, which does not ensure that the questions accurately represent the information needs of real users. In contrast, our collection has been collected over time by different KG maintainers across the SIB, where most examples reflect questions asked by real users of the data.
3. Small scale. The higher-complexity, human-written question–query collections, such as the QALD series (Question Answering over Linked Data challenge updated each year, e.g., [52]), only include a small number of queries. Datasets which have been proposed for benchmarking the performance of federated queries specifically similarly only include a small number of queries. As an example, LargeRDFBench [53] includes only 40 (federated-only) queries of varying complexity. Similarly, FedBench [51] is a very good resource for benchmarking federated queries (including federated queries joining more target endpoints than our collection, i.e., up to 16 sources); however, there are only 36 queries in total. In contrast, our dataset includes over 1,000 question–query pairs, all of which have been human-written and collected over time from the different KG maintainers.
4. HAMAP as SPARQL [54] is also a collection of more than 4,000 queries for a specific goal, annotating proteins. However, these are not aimed at educating users to the capabilities of a system. Nor are they annotated with a text for what their purpose is.
5. SPIN [55], SPARQL Inference Notation, is a W3C member submission that informed some of the design decisions of SHACL. SPIN provides for SPARQL templates and has logic for describing arguments to queries. However, its focus on constraints leads its modeling logic to ignore the concept of using these queries to mark up examples.
6. The Wikidata project has a media wiki template [56] for SPARQL queries. However, this does not standardize the metadata about the queries, nor does it provide the necessary utilities to help users maintain their queries.

A systematic overview and comparison of federated query benchmarks is provided in [57]. However, the overview focuses on the performance aspect of federated query processing, as opposed to natural language question-to-query translation.

## Conclusion

In this paper, we introduce a large collection of example questions and their corresponding SPARQL queries collected over time across the catalog of SIB KGs and beyond. Moreover, we have proposed a methodology to standardize the representation of these examples, facilitating their uniform processing by both users and services. Finally, we have presented a comprehensive portfolio of services that leverage the standardization of metadata across the collection of examples, including services for the automated testing, editing, and visualizing of example queries. All of these are available open-source and can easily be adapted by other KG maintainers for their own purposes, provided that maintainers adopt our recommended approach.

In the future, we plan to extend the current methodology to support defining templates in the example queries, in other words, fields that can be dynamically filled by querying a dedicated API (e.g., a SPARQL query). This would be a generalization of our current format, avoiding hard-coding literals in the example queries. We also plan to deploy an LLM-based solution to automatically translate user questions in natural language to machine-readable SPARQL queries, leveraging the collection presented here.

We encourage the community to contribute to our proposed metadata specification, towards FAIRer KGs, which will pave the way for improved Semantic Web tools. The advent of new technologies, such as large language models, provides an opportunity for enabling a wider audience, including researchers and the public at large, to fully benefit from the wealth of interconnected data available in distributed KGs. We believe that a set of representative metadata standards for KGs, including the SPARQL examples metadata proposed here, represent an important and pragmatic step forward in this direction.

## Abbreviations

DBGI: Digital Botanical Gardens Initiative; FAIR: findable, accessible, interoperable, reusable; HTTP: Hypertext Transfer Protocol; KG: knowledge graph; KGQA: knowledge graph question answering system; LLM: large language model; QALD: Question Answering over Linked Data; RDF: Resource Description Framework; RDFS: RDF Schema; SHACL: Shapes Constraint Language; SIB: Swiss Institute of Bioinformatics; SPARQL: SPARQL Protocol and RDF Query Language; TTL: Terse RDF Triple Language; VoID: Vocabulary of Interlinked Datasets.

## Supplementary Material

giaf045_Authors_Response_To_Reviewer_Comments_Original_Submission

giaf045_GIGA-D-24-00433_Original_Submission

giaf045_GIGA-D-24-00433_Revision_1

giaf045_Reviewer_1_Report_Original_Submission Alban Gaignard -- 11/7/2024

giaf045_Reviewer_1_Report_Revision_1 Alban Gaignard -- 3/11/2025

giaf045_Reviewer_2_Report_Original_Submission Rita Sousa -- 12/4/2024

## Data Availability

The catalog of examples is available via GitHub [13]. A set of tools to convert, test, and help maintain SPARQL query examples is available via GitHub [26]. An archival copy of the example catalog is also available via the GigaScience database, GigaDB [58]. Associated codes are licensed under the MIT license and sparql queries under dual licenses MIT and CC-BY-4.0. All referenced datasets are publicly available, while the services leveraging the examples are all available open-source. The links to each of these are provided as references in the article text.
