# Supplementary material for: A large collection of bioinformatics question–query pairs over federated knowledge graphs: methodology and applications
Source: Gigascience. 2025 May 16;14:giaf045. doi: 10.1093/gigascience/giaf045 (PMC12083453; doi:10.1093/gigascience/giaf045)
Supplement: giaf045_GIGA-D-24-00433_Revision_1 [file giaf045_giga-d-24-00433_revision_1.pdf]

GigaScience

A large collection of bioinformatics question-query pairs over federated knowledge graphs: methodology and applications

--Manuscript Draft--

**Manuscript Number:**	GIGA-D-24-00433R1
**Full Title:**	A large collection of bioinformatics question-query pairs over federated knowledge graphs: methodology and applications
**Article Type:**	Technical Note

Funding Information:

Schweizerischer Nationalfonds zur Förderung der Wissenschaftlichen Forschung (20CH21_217482)	Dr. Ana Claudia Sima
National Human Genome Research Institute	Dr. Alan Bridge
Division of Microbiology and Infectious Diseases, National Institute of Allergy and Infectious Diseases	Dr. Alan Bridge
National Institute on Aging	Dr. Alan Bridge
National Institute of General Medical Sciences	Dr. Alan Bridge
National Institute of Diabetes and Digestive and Kidney Diseases	Dr. Alan Bridge
National Eye Institute	Dr. Alan Bridge
National Cancer Institute (US)	Dr. Alan Bridge
National Heart, Lung, and Blood Institute (U24HG007822)	Dr. Alan Bridge
State Secretariat for Education, Research and Innovation SERI (CH)	Ms. Severine Duvaud
swissuniversities (Swiss Open Research Data Grants (CHORD) in Open Science I, grant id “Swiss DBGI-KM”)	Dr. Tarcisio Mendes de Farias
State Secretariat for Education, Research and Innovation SERI (CH) (101188078 — FIDELIS)	Ms. Severine Duvaud

Abstract:

Background

In the last decades, several life science resources have structured data using the same framework and made these accessible using the same query language to facilitate interoperability. Knowledge graphs have seen increased adoption in bioinformatics due to their advantages for representing data in a generic graph format. For example, yummydata.org catalogs more than 60 knowledge graphs accessible through SPARQL, a technical query language. Although SPARQL allows powerful, expressive queries, even across physically distributed knowledge graphs, formulating such queries is a challenge for most users. Therefore, to guide users in retrieving the relevant data, many of these resources provide representative examples. These examples can also be an important source of information for machine learning, if a sufficiently large number of examples are provided and published in a common, machine-readable and standardized format across different resources.

Findings

We introduce a large collection of human-written natural language questions and their corresponding SPARQL queries over federated bioinformatics knowledge graphs (KGs) collected for several years across different research groups at the SIB Swiss Institute of Bioinformatics. The collection comprises more than 1000 example questions and queries, including 65 federated queries. We propose a methodology to uniformly represent the examples with minimal metadata, based on existing standards.

Powered by Editorial Manager® and ProduXion Manager® from Aries Systems Corporation

	Furthermore, we introduce an extensive set of open-source applications, including query graph visualizations and smart query editors, easily reusable by KG maintainers who adopt the proposed methodology.<br><br>Conclusions<br><br>We encourage the community to adopt and extend the proposed methodology, towards richer KG metadata and improved Semantic Web services.<br><br>URL<br><br>https://github.com/sib-swiss/sparql-examples
**Corresponding Author:**	Ana Claudia Sima, Ph.D.<br>SIB: Swiss Institute of Bioinformatics<br>Lausanne, SWITZERLAND
**Corresponding Author Secondary Information:**	
**Corresponding Author's Institution:**	SIB: Swiss Institute of Bioinformatics
**Corresponding Author's Secondary Institution:**	
**First Author:**	Jerven Bolleman
**First Author Secondary Information:**	
**Order of Authors:**	Jerven Bolleman
	Vincent Emonet
	Adrian Altenhoff
	Amos Bairoch
	Marie-Claude Blatter
	Alan Bridge
	Severine Duvaud
	Elisabeth Gasteiger
	Dmitry Kuznetsov
	Sébastien Moretti
	Pierre-Andre Michel
	Anne Morgat
	Marco Pagni
	Nicole Redaschi
	Monique Zahn-Zabal
	Tarcisio Mendes de Farias
	Ana Claudia Sima, Ph.D.
**Order of Authors Secondary Information:**	
**Response to Reviewers:**	Dear Editors and Reviewers,<br><br>We would like to thank the reviewers for all their insightful comments, improvements and suggestions and the editors for considering our article for a possible publication in GigaScience.<br><br>We have revised the paper in order to take all comments into account. We thus hope that this new version of our article complies with the remarks and answers the

questions raised by the reviewers.

The following paragraphs list point-by-point the issues identified by the reviewers, followed by our corresponding answers. Finally, to facilitate the review process, the modified and added sentences are highlighted in red in the revised version of our article.

NOTE: due to journal formatting requirements, all links and footnotes in the original submission have been replaced, throughout the manuscript, with references.

Reviewer #1:
REVIEWER:Targeting the life science community, the authors introduce a very valuable resource, aimed at facilitating the sharing and reuse of data through public knowledge graphs. The paper is extensive and very interesting, with many technical details. Although it is well-illustrated, it is not always easy to follow and I have some concerns.
AUTHORS: Thank you for this comment. We hope the revised version addresses all concerns pointed out.

REVIEWER: The readability of the paper could be improved with a better introduction for some of the standards and technologies. For example, SHACL or VoID could be better described, and their use more clearly justified.
AUTHORS: We have added more clarifications on these in the “Implementation” section.

REVIEWER: Moreover, the connection between the applications and the objectives of the paper is not always very clear. How does each of the proposed applications, in conjunction with the proposed query catalog, contribute to the challenges identified in the introduction?
AUTHORS: Thank you for raising this point, we have provided more clarifications in the Application section.

REVIEWER: Finally, the thousand queries, which are the core of the contribution, are not precisely, and quantitatively described. How are they representative of the content of the knowledge graphs ? Which parts of the ontologies are covered by the queries (used properties, classes) ?
AUTHORS: We provide a script to automatically compute coverage in our GitHub repo https://github.com/sib-swiss/sparql-llm/blob/main/notebooks/compare_queries_examples_to_void.ipynb . The script helps to provide an overview of which classes and predicates are not covered by the examples.
However, this metric is also sometimes tricky to automate - consider the case where types are not explicitly defined in the queries - in this case, it can be hard to automatically infer the class (i.e. the rdf:type) of variables from the query text. Therefore, the coverage numbers reported by the script must be considered only as approximate. To the best of our knowledge, coverage is rarely (if ever) reported in published literature even of official benchmarks for natural language to query translation (such as QALD or LC-QUAD). For additional metrics regarding the complexity of our queries please also see https://sib-swiss.github.io/sparql-examples/examples/algebra-statistics.html .

REVIEWER: How many results are returned by the queries ?
AUTHORS: We thank the reviewer for the question. The main reason why we decided to not provide the number of query results is that the results may change overtime according to new releases of the RDF datasets, for example, UniProt regularly updates its knowledge graph on average every 3 months. Moreover, some of the queries have limits to assure that an example runs quickly, including subqueries with limits that may change the result count on different executions of the same SPARQL query. Our main focus is on the translation of natural language questions to their equivalent queries (and indeed we expect this to be more stable over time, as schema changes are more infrequent than content-level updates).

REVIEWER:Queries are proposed by the data providers, but are they similar or

Powered by Editorial Manager® and ProduXion Manager® from Aries Systems Corporation

different from the actual served queries (SPARQL endpoint query logs) ?
AUTHORS: Some examples are inspired by query logs, however, we only have limited access to these logs in the first place, due to Swiss federal law similar to GDPR. Moreover, this is out of the scope of this work since it will require not only to analyse the individual logged queries compared to our example query set but also guess or predict the corresponding natural language translation by capturing the user intent when formulating a SPARQL federated query. In addition, we are dealing with highly specialized scientific questions that can require cross-domain expertise, not necessarily covered by the authors' competencies.
We will ask all authors

REVIEWER: Is it possible to quantify typical SPARQL patterns (property paths, aggregates, etc.) ?
AUTHORS: Yes, we can count the usage of basic SPARQL features, and we now include a markdown report of such in our output when converting the examples to Markdown. This full report can be found at https://sib-swiss.github.io/sparql-examples/examples/algebra-statistics .

REVIEWER: Regarding the discussion, the limits of the approach are not described. Nothing is said regarding the evolution of the knowledge graphs, both in terms of schema and content, and the costs of maintaining the query catalog up-to-date.
AUTHORS: The costs of maintaining the catalog up-to-date are split among the SPARQL endpoint maintainers. In the future, we could add more tests (that some of our teams already do) to check for queries that fail (or that return empty results where this is not expected). However, currently each KG maintainer is responsible for keeping their queries up-to-date.

REVIEWER: Apart from that, the proposed resource and its sharing with the broader community is very timely and essential, given the recent advances and extremely active research on LLMs.
AUTHORS: Thank you very much for the positive response!

Introduction
REVIEWER: "High quality" : what do you mean by "high-quality ? This would be useful to evaluate the proposed query catalog.
AUTHORS: Our specific angle is on realistic, non-synthetic questions that reflect the information needs of real users. Although this is a harder quality to evaluate automatically, we believe that having queries written by endpoint maintainers to support their users should result in higher quality queries, rather than those (semi-)automatically generated for benchmarking purposes (e.g. LC-Quad 1 and LC-Quad 2).

REVIEWER: "Representative" : same question, which leads to "how the proposed questions are representative with respect to the 11 knowledge graphs ?"
AUTHORS: We refer the reviewer to our response for the previous question - since endpoint maintainers themselves provide the examples (and they are the most knowledgeable authority with respect to their own data, while also having a direct interest in covering user information needs with the provided examples), we believe this results in the representativeness of the queries.

REVIEWER: "The rapid development and uptake of these services" could you provide evidence for that ? Do user issue SPARQL queries ? Are SPARQL queries wrapped through web APIs ? Generated by algorithms ?
AUTHORS: Across SIB SPARQL endpoints, we have both services and users that issue queries, with varying degrees of uptake (ranging from thousands of unique queries per month in the case of UniProt to a few tens for smaller KGs). All SPARQL endpoints described in the paper provide a webpage which enables direct querying with federated SPARQL 1.1 support. We have also started preliminary work to automatically generate R, Java and Python APIs to enable programmatic access to the SPARQL endpoints (e.g., https://github.com/TRIPLE-CHIST-ERA/sparql-api-codegen), but since this work is not mature enough we did not include it in this article. Moreover, Web APIs can also be generated by using existing tools like "grlc" (see https://github.com/CLARIAH/grlc) as pointed out in our git repository https://github.com/sib-swiss/sparql-examples?tab=readme-ov-file#generate-rq-files .
We will ask all authors

REVIEWER: A clarification is needed about the 10 or 11 SIB KGs ? (Introduction VS Sources sections)
AUTHORS: Thank you for pointing out this mismatch - indeed the information in the Sources is the correct one (10 KGs are SIB's, 1 is the result of an external collaboration)

Sources
REVIEWER: "source of information for machine learning": it's a bit vague, what would be the targeted ML tasks ?
AUTHORS: Thank you for the observation. Indeed, some of the machine learning tasks that would be possible are in the realm of translating natural language questions to SPARQL across heterogeneous Knowledge Graphs. We have added a specific mention of this now in the text.

REVIEWER: "… complexity of the queries": I'm not convinced by counting the average of triple patterns as an estimate of the complexity. What about the use of Unions, Negation, Service clauses ? The number of used variables, the use of property paths, etc. ?
AUTHORS: For some more detailed statistics we have now made available a script that counts automatically e.g. the number of aggregations, Service clauses etc. across the catalog of queries. The output of this script is available at https://sib-swiss.github.io/sparql-examples/examples/algebra-statistics.

REVIEWER: "small KG": a KG can also be large if you consider the number of class instances … or properties … not only the size of the schema.
AUTHORS: Thank you for this point, we have rephrased to explicit mention the size of the schema.

REVIEWER: "average number of TP per endpoint": to be clarified, is it per endpoint ? Is it per query ?
AUTHORS: Many thanks for this observation, indeed it is the average number of triple patterns per query, for each endpoint. We have added this clarification in the text.

Methodology
REVIEWER: I could not find anything on the SHACL spec about SPARQLExecutable concepts. As it is presented in the paper, it is unclear if it's something new or part of the spec. If it is introduced by the authors, it should be better described and motivated.
AUTHORS: Note that although SHACL is documented as a technical report and a W3C recommendation in (https://www.w3.org/TR/shacl/), the vocabulary terms we use are only fully described in its RDF serialization, that is either Turtle or RDF/XML in (https://www.w3.org/ns/shacl). We added this explanation in the "Implementation" section for clarity, and updated the links/references in the paper to refer directly to the SHACL vocabulary in turtle.

REVIEWER: "aggregate information about the collection of examples": Why ? How ?
We have added an example in the text and a clarification regarding where more specific queries can be found in "Querying for queries" in the GitHub repo. More aggregations, such as COUNT all queries that target a specific endpoint, are possible via similar mechanisms.

REVIEWER: "We provide a script": where ?
AUTHORS: The script can be found in the GitHub repository, section "Compile all query files into one file to upload to your endpoint".

REVIEWER: Listing 2: is it only possible to do "keyword-search" ? If so, this should be part of the discussion of the paper
AUTHORS: The user can have a better local "indexing" of the examples, leveraging their uniform representation. For example, users could upload examples into a vector search engine locally. Listing 2 is just a simple illustration to show that example queries can themselves be queried as any other RDF data, including for search purposes.

Powered by Editorial Manager® and ProduXion Manager® from Aries Systems Corporation

REVIEWER: VoID (and SHACL) should be better introduced/described
AUTHORS: Indeed, we have added more details in the “Implementation” section.

Applications
REVIEWER: The paragraphs on "automated testing" and "fixing SPARQL 1.1 syntax" should be better aligned with the main objectives of the paper
AUTHORS: The two paragraphs illustrate how automated testing can help support endpoint maintainers to keep the catalog of example queries up-to-date, which is in line with providing a catalog of high-quality, up-to-date representative queries for endpoint users (which is the goal of the paper).

REVIEWER: "decomposing graph paths": why not speaking about SPARQL property paths ?
AUTHORS: Done.

REVIEWER: The visualization of the BGP seems to be very useful for non SPARQL experts, congratulations !
REVIEWER: Query editor: how does it differ from Qlever auto-completion ?
AUTHORS: The Qlever UI query editor is not triplestore agnostic as compared to our approach. Although the autocomplete method proposed in [1] by the Qlever team is described to be applicable to other triple stores, the code is not publicly available anymore. Moreover, as stated in [2], the Qlever UI query editor is “a user-friendly interface for the Qlever SPARQL engine”, hence, it is not triple-store agnostic.
We acknowledge that a combination of both (our approach and Qlever) could provide overall even better results to the users, while remaining triplestore agnostic, therefore indeed we could foresee a collaboration with Qlever on this in the future.
We have added a discussion on this in the corresponding “Query editing and searching” section of the paper.

[1] https://ad-publications.cs.uni-freiburg.de/CIKM_sparql_autocompletion_BKKKS_2022.pdf
[2] https://github.com/ad-freiburg/qlever-ui?tab=readme-ov-file

REVIEWER: Bio-query : it is not totally clear how this example application supports the paper. What, in the methodology, allows to better categorize the 1000 queries in BioQuery ?
AUTHORS: The integration in BioQuery is shown as an example of the benefits of uniform SPARQL question-query metadata representation with respect to the services that can consume these examples. Representing all examples uniformly across different endpoints has facilitated their direct integration (and searchability) in the BioQuery interface.
In the future, we plan to add specific metadata to enable queries to be “templated” in the collection of examples, such that they can also be made configurable in the BioQuery interface (e.g. allowing users to replace specific entities in the questions, such as species or gene names, making the questions more easily adaptable to the needs of the users without requiring SPARQL knowledge, e.g. replacing “human” or “TP53” in “What are the orthologs of the human TP53 gene in the rat?” with the appropriate species / gene name).

Discussion
REVIEWER: "… to triples that do not exist": we understand implicitly that the query will not return any result, but it should be made explicit
AUTHORS: We have added the explicit mention in the text.

REVIEWER: The paragraph on "follow-up" questions is unclear
AUTHORS: We have rephrased this for clarity to “These questions would need to be rephrased such that they are self-contained, as most existing ML systems for translating natural language questions to queries treat each question in the collection as independent of the others.”

Related works
REVIEWER: In terms of structure, it seems more natural to describe related works before the discussion.
AUTHORS: The placement of “Related Works” is oftentimes dependent on the

Powered by Editorial Manager® and ProduXion Manager® from Aries Systems Corporation

common practice in the community or discipline - in our work, we have opted to leave this towards the end, favoring the introduction of our specific sources and methodology first.

REVIEWER: Some federated sparql query benchmarks should be better described in the related works such as at least FedBench, or LargeRDFBench ([dice-group/LargeRDFBench: LargeRDFBench: A Billion Triples Benchmark for SPARQL Query Federation](https://github.com/dice-group/LargeRDFBench?tab=readme-ov-file#benchmark-queries)), where the natural language question is given for instance "Return for all US presidents their party membership and news pages about them."
AUTHORS: Both FedBench and LargeRDFBench are good references for benchmarking federated query performance, however for the purposes of natural language question to query translation they both might be small, as they each include only about 40 queries pairs (i.e. the “large” aspect in LargeRDFBench indicates the size of the composing sources, which aggregate to over 1B triples, not the size of the query collection). We have added a description on both LargeRDFBench and FedBench in the Related Work.

Reviewer #2:
REVIEWER: Overall, this paper presents valuable resources that can benefit not only the semantic web community but also a broader audience of KG users. However, I believe certain aspects of the paper's organization and clarity could be enhanced. Below, I leave some suggestions:
AUTHORS: The authors thank the reviewer for supporting our work. We hope this revised version addresses all reviewers’ concerns.

REVIEWER: In the "Sources" section, I missed a brief description of the 10 KGs to understand better the different domains covered in the queries. Additionally, detailed statistics on the 1000 queries, such as the distribution of queries per KG and the proportion of federated queries, seem to be missing.
AUTHORS: For a description of the domains of the SIB KGs, we refer the reader to the NAR paper [1]. We have added more detailed statistics on the number of federated queries and other query characteristics online at https://sib-swiss.github.io/sparql-examples/examples/algebra-statistics.

[1] "The SIB Swiss Institute of Bioinformatics Semantic Web of data." Nucleic Acids Research 52, no. D1 (2024): D44-D51, https://doi.org/10.1093/nar/gkad902 .

REVIEWER: In the "Implementation" section, alongside the example of a SELECT query, it would be beneficial to include an example of a DESCRIBE query since new properties and concepts have been introduced to represent this query type.
AUTHORS: We have added a link to a concrete example, as well as a description of the specific metadata required in such cases, in the Implementation section.

REVIEWER: I think the organization of the "Application" section and the following subsections could be improved. The initial list of possible applications does not align with the structure of the subsequent subsections, leading to some confusion.
AUTHORS: Thank you for raising this point, we have changed the order of the initial list in the Application section to conform with the subsequent sections.

REVIEWER: In the "Query editing and searching" section, adding a figure similar to the one provided in the "Integration in Bio-Query" section to illustrate the features would enhance clarity.
AUTHORS: We thank the reviewer for the suggestion, we have now integrated a screenshot in the section.

REVIEWER: In the "Related Work" section, including a summarizing table where existing collections are listed in rows and evaluated against characteristics such as scale, the inclusion of federated queries, representativeness, etc., would provide a clear comparison.
AUTHORS: We have provided a more systematic overview in the Related Work section, including a federated benchmark survey paper. However, our goal is not necessarily to survey and compare with existing benchmarks, but to provide a realistic use case and collection of questions and queries collected over time within 11 publicly

	available bioinformatics SPARQL endpoints.<br><br>Minor Comments:<br>REVIEWER: On page 5, the sentence: "For example, the query defined at https://www.bgee.org/sparql/.well-known/sparqlexamples/1 could directly reference Listings 1.<br>Indeed, we have now added this reference.<br><br>REVIEWER: In the caption of Figure 3, replace the word "here" with the actual URL to ensure the link is explicit and accessible.<br>AUTHORS: Thank you for the suggestion, indeed the formatting has now been revised to explicitly add all URLs as references (at the end of the paper).
**Additional Information:**	
**Question**	**Response**
Are you submitting this manuscript to a special series or article collection?	No
**Experimental design and statistics**<br><br>Full details of the experimental design and statistical methods used should be given in the Methods section, as detailed in our Minimum Standards Reporting Checklist. Information essential to interpreting the data presented should be made available in the figure legends.<br><br>Have you included all the information requested in your manuscript?	Yes
**Resources**<br><br>A description of all resources used, including antibodies, cell lines, animals and software tools, with enough information to allow them to be uniquely identified, should be included in the Methods section. Authors are strongly encouraged to cite Research Resource Identifiers (RRIDs) for antibodies, model organisms and tools, where possible.<br><br>Have you included the information requested as detailed in our Minimum Standards Reporting Checklist?	Yes
**Availability of data and materials**	Yes

Powered by Editorial Manager® and ProduXion Manager® from Aries Systems Corporation

All datasets and code on which the conclusions of the paper rely must be either included in your submission or deposited in publicly available repositories (where available and ethically appropriate), referencing such data using a unique identifier in the references and in the "Availability of Data and Materials" section of your manuscript.<br><br>Have you have met the above requirement as detailed in our Minimum Standards Reporting Checklist?	

Manuscript

Click here to access/download;Manuscript;SPARQL-query examples_for_production.docx

A large collection of bioinformatics question-query pairs over federated knowledge graphs: methodology and applications

Jerven Bolleman[1,*] https://orcid.org/0000-0002-7449-1266,
Vincent Emonet[1,*] https://orcid.org/0000-0002-1501-1082,
Adrian Altenhoff[1] https://orcid.org/0000-0001-7492-1273,
Amos Bairoch[1] https://orcid.org/0000-0003-2826-6444,
Marie-Claude Blatter[1] https://orcid.org/0000-0002-7474-1499,
Alan Bridge[1] https://orcid.org/0000-0003-2148-9135,
Séverine Duvaud[1] https://orcid.org/0000-0001-7892-9678,
Elisabeth Gasteiger[1] https://orcid.org/0000-0003-1829-162X,
Dmitry Kuznetsov[1] https://orcid.org/0000-0002-9972-947X,
Sébastien Moretti[1] https://orcid.org/0000-0003-3947-488X,
Pierre-Andre Michel[1] https://orcid.org/0000-0002-7023-1045,
Anne Morgat[1] https://orcid.org/0000-0002-1216-2969,
Marco Pagni[1] https://orcid.org/0000-0001-9292-9463,
Nicole Redaschi[1] https://orcid.org/0000-0001-8890-2268,
Monique Zahn-Zabal[1] https://orcid.org/0000-0001-7961-6091,
Tarcisio Mendes de Farias[1,†] https://orcid.org/0000-0002-3175-5372,
Ana Claudia Sima[1,†] https://orcid.org/0000-0003-3213-4495

[1] *SIB Swiss Institute of Bioinformatics, Switzerland*
Correspondence address: Tarcisio Mendes de Farias, Ana Claudia Sima, SIB Swiss Institute of Bioinformatics, Amphipôle, Quartier UNIL-Sorge, 1015 Lausanne, Switzerland e-mail: tarcisio.mendes@sib.swiss, ana-claudia.sima@sib.swiss

Abstract

Background. In the last decades, several life science resources have structured data using the same framework and made these accessible using the same query language to facilitate interoperability. Knowledge graphs have seen increased adoption in bioinformatics due to their advantages for representing data in a generic graph format. For example, yummydata.org catalogs more than 60 knowledge graphs accessible through SPARQL, a technical query language. Although SPARQL allows powerful, expressive queries, even across physically distributed knowledge graphs, formulating such queries is a challenge for most users. Therefore, to guide users in retrieving the relevant data, many of these resources provide representative examples. These examples can also be an important source of information for machine learning (for example, ML algorithms for translating natural language questions to SPARQL), if a sufficiently large number of examples are provided and published in a common, machine-readable and standardized format across different resources.
Findings. We introduce a large collection of human-written natural language questions and their corresponding SPARQL queries over federated bioinformatics knowledge graphs (KGs) collected for several years across different research groups at the SIB Swiss Institute of

*co-first authors
[†] corresponding author

Bioinformatics. The collection comprises more than 1000 example questions and queries, including almost 100 federated queries. We propose a methodology to uniformly represent the examples with minimal metadata, based on existing standards. Furthermore, we introduce an extensive set of open-source applications, including query graph visualizations and smart query editors, easily reusable by KG maintainers who adopt the proposed methodology.
Conclusions. We encourage the community to adopt and extend the proposed methodology, towards richer KG metadata and improved Semantic Web services.
URL: https://github.com/sib-swiss/sparql-examples

Keywords: knowledge graphs, Resource Description Framework (RDF), federated SPARQL, query editor, metadata

Introduction

The accuracy and real-world applicability of question answering systems over structured data crucially depend on the availability of high-quality, representative question-query pairs over diverse datasets. Resources, such as UniProt [1], have been used in bioinformatics for many years, where they facilitate data integration and exploration through (potentially federated) SPARQL queries. However, a long-standing challenge is making this wealth of data usable by a wider range of users, including researchers without technical training. Writing SPARQL queries is outside the competence of most life scientists, as it requires both knowledge of the query language itself and understanding of the knowledge graph schema, for example, a Resource Description Framework (RDF) graph data model. Many solutions have thus been investigated, including Natural Language Interfaces (NLI) to knowledge graphs (KG). The recent progress in Large Language Models (LLMs) has enabled to boost performance across a wide range of Natural Language Processing tasks, including promising results in LLM-based Knowledge Graph Question Answering (KGQA) systems [2]. A recent study highlights that unified models, trained across diverse datasets stemming from distinct knowledge graphs, eliminate the need for separate models for each KG and can therefore significantly reduce the overall costs of fine-tuning and deploying such models [3]. Therefore, a key requirement to the development of novel, LLM-based KGQA systems, is the availability of high-quality, representative public datasets of questions and corresponding SPARQL queries across a wealth of diverse KGs.

Capturing user intent and translating questions becomes significantly more complex when considering multiple, distributed scientific KGs such as those comprising the SIB Swiss Institute of Bioinformatics Semantic Web of Data. Although federated SPARQL queries are very powerful because they enable joint querying of physically distributed knowledge graphs, the challenge lies in formulating such queries. Federated queries are inherently complex because they require users to not only understand the data models of the individual sources involved in the query, but also to be aware of how these sources interconnect (*i.e.*, how they can be joined). Hence, the availability of examples to guide users - or of a system supporting the automatic translation of user questions to federated queries - are critical. However, federated queries have been mostly neglected so far in existing collections of natural language questions and equivalent SPARQL queries.

The SIB hosts a number of high-quality, curated and interoperable life science knowledge graphs [4] that are accessible via public SPARQL endpoints, i.e. web addresses that can receive and process SPARQL queries. Each of these KGs is independently managed by different research groups within the institute. However, a common point across all resources is that they each maintain a set of representative questions and their equivalent SPARQL queries, collected over time. These are made available to inspire and help users formulate new queries over the respective KG data. Importantly, the resources also include examples of federated queries, which allow for joining information from their KG with those of other, complementary KGs, from the SIB catalog and beyond (e.g., Wikidata [5], IDSM [6], ChEMBL [7]). Prior to the work presented in this paper, the examples were provided in heterogeneous and sometimes non-machine-readable formats (e.g., as plain text in a static webpage). However, to facilitate interoperability and more generally the reusability of all examples as a large, uniform collection, it is of paramount importance to standardize how queries are represented, in agreement with the FAIR data principles (Findable, Accessible, Interoperable and Reusable), allowing their automatic discoverability by both users and services.

In this paper, we introduce the growing collection of over 1,000 human-written, real-world question-query pairs, including almost 100 federated queries (i.e., queries that ask questions requiring at least 2 sources jointly), collected over several years across 11 interoperable knowledge graphs. Additionally, we describe the methodology we have applied across SIB to represent, store and test example queries by adopting different (meta)data standards. Importantly, we show that this standardization enables easily finding examples across different endpoints, as well as setting up, running, and maintaining services that use these examples. These services include powerful user-facing applications, such as search engines or automatically generated web visualizations. The rapid development and uptake of these services across different SIB Resources directly prove the value of adopting FAIR data principles and metadata standardization. To our knowledge, ours is the most comprehensive portfolio of bioinformatics examples and services facilitated by FAIR KG (meta)data across distributed KGs. Both the collection of example questions-SPARQL queries, as well as all the developed services leveraging these examples, are available open-source and can be adapted to other KGs by following our recommended metadata format.

We encourage the community to engage with the collection of examples, adopt and further extend our proposed format for describing SPARQL examples, and finally reuse and improve the services presented in this work. The SIB community will continue updating the catalog with new examples. Efforts towards standardization will not only contribute towards FAIRer Knowledge Graphs, but will also help build a solid foundation for a new generation of Semantic Web Technologies, such as LLM-based Natural Language Interfaces over Knowledge Graphs.

Sources

At the time of writing, we have collected contributions from 10 large-scale KGs across different SIB groups. Additionally, we integrated contributions from one external KG, "*dbgi*", with examples contributed by external collaborators from the Digital Botanical Gardens Initiative [8]. The collection is continuously updated with new examples. An up-to-date overview of the number of queries, including the number of federated queries, is generated automatically from the collection and can be visualized live online [9].

For details on the SPARQL endpoints maintained by the SIB, we refer the reader to the SIB Semantic Web of Data [10]. Among the contributing KGs, we can mention UniProt - currently the largest publicly available KG - with over 168B triples and a total of 61 contributed example queries. To our knowledge, this collection is the most comprehensive collection of real-world SPARQL queries examples to-date in bioinformatics.

As an estimate of the complexity of the queries in this collection, we show the average number of triple patterns (TP) per query for each endpoint, included in the collection in Figure 1. The number of TPs is a good approximation for the complexity of a query, because it indicates the number of hops (joins) required to compute an answer over the graph. Most queries have around 6 TPs on average, with the notable exception of the DBGI, for which queries have a much higher complexity, owing to the complexity of the data model, but also due to federation with Wikidata, which is present in most examples. On the other end, HAMAP includes only a few very simple examples, as it has a relatively small schema (encompassing only 7 classes) for describing annotation rules. We note that most existing large scale collections focus on simple queries, i.e. queries with at most 3 TPs (for examples, see Section Related Work). Additional information on query complexity (for example, total number of aggregations, like SUM, COUNT etc.) is generated automatically from the catalog and available online [9].

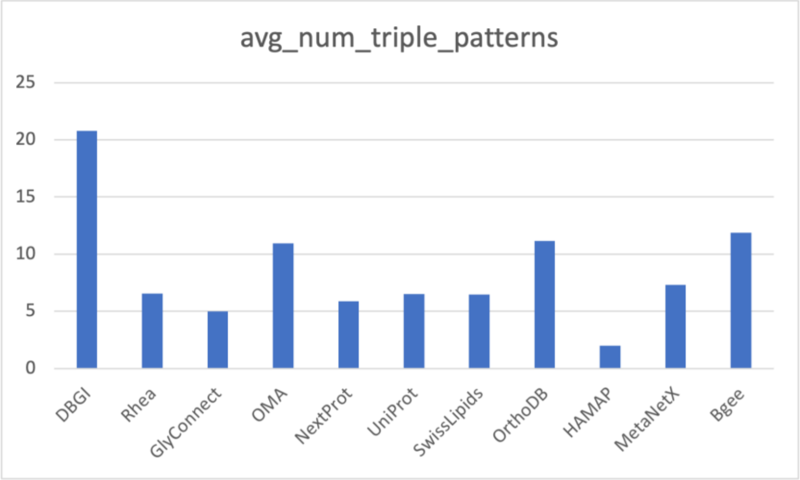

Figure 1. Average number of triple patterns per query in each of the KGs contributing to the examples collection

Methodology

Standardization is at the core of our approach. To structure and describe the question-query pairs, we rely on the SHapes Constraint Language (SHACL) vocabulary, which is RDF based [11]. In addition to SHACL and RDF Schema (RDFS) [12], we describe and annotate the examples with Schema.org. Standardizing how we represent the examples is crucial since they can target different and independent data sources managed by distinct groups and organizations. A standardized, machine-readable representation, enables both users and services to discover and smoothly use the question-query descriptions that are defined in RDF and stored in distributed data stores accessible with SPARQL. More precisely, example question-query pairs can be stored with their associated metadata in the same RDF store, consequently, making them accessible via the same SPARQL endpoint. As a result, this standardization and infrastructure facilitate the automatic testing of queries, as well as the deployment of services with minimal code modifications across endpoints, such as the generation of user-friendly Web pages with query descriptions and graphical visualizations (see Section Applications).

Figure 2 illustrates the end-to-end workflow, starting from examples contributed by KG maintainers to services consuming the examples from the named graph they are published in.

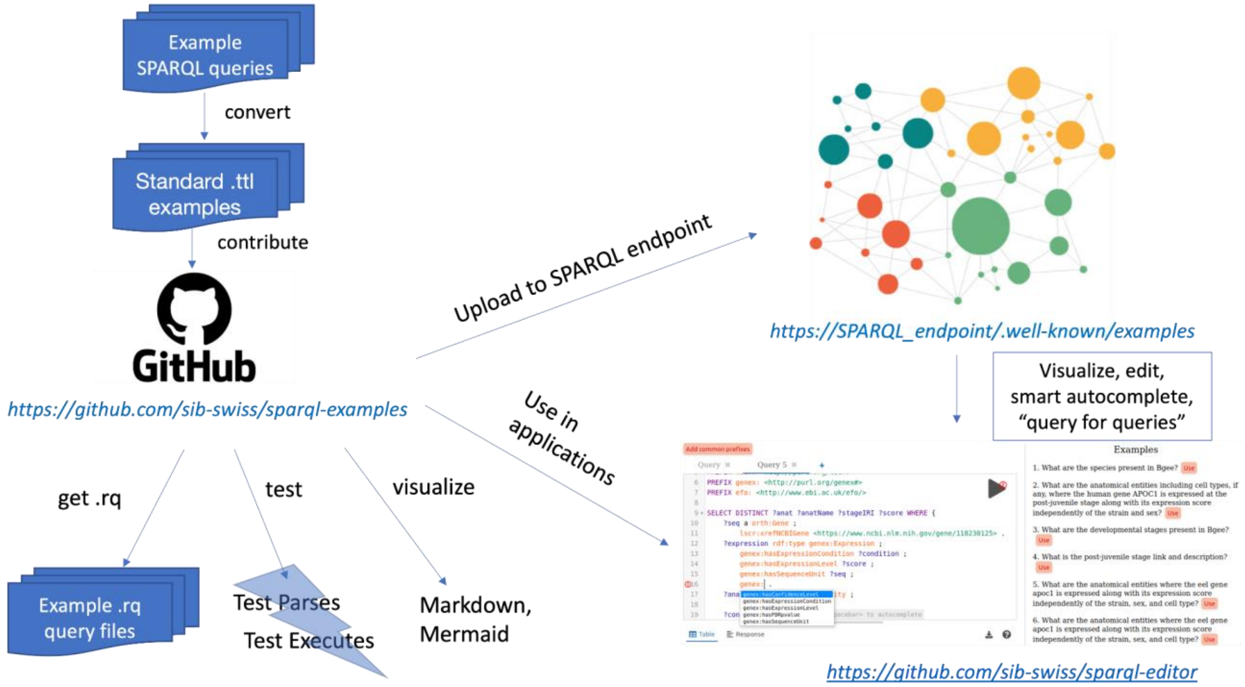

Figure 2. End-to-end workflow, from example contributions to services that uniformly consume examples across distinct SPARQL endpoint (e.g. sparql-editor and Bio-Query template search)

Implementation

We collaborate in an open GitHub repository [13] . The repository is the "single source of truth", where all SPARQL examples are collected via contributions from data store maintainers. Each

example is stored in a separate file using the Terse RDF Triple Language (Turtle, *i.e.* ".ttl") syntax and file format [14], within a folder named according to the resource that contributed the example. For example, all Bgee queries can be found in [15] .

Each Turtle file stores a single example, which is represented by:

a) The type of the query (e.g., Ask, Select, Describe).
b) The description of the query (e.g., the question in plain text), including a language tag.
c) The SPARQL query itself.
d) The target SPARQL endpoint(s) where the query can be executed.
e) Additional keywords (tags).

Each question-query pair is defined as a *SPARQLExecutable* SHACL concept [11]. Note that although SHACL is documented as a technical report and a W3C recommendation in [16], the vocabulary terms we use are only fully described in its RDF serialization either Turtle or RDF/XML in [17]. We also specify the *SPARQLExecutable* subtype, such as *SPARQLAskExecutable* and *SPARQLSelectExecutable*, that is executable queries based on an *ASK* and *SELECT SPARQL* query, respectively. SHACL as standard is aimed at describing shapes that data may conform to. These shapes may then be used for validation, user interfaces and optimizations. We use the SHACL standard as each SPARQL query can be seen as a declaration of a data shape that a SPARQL engine instantiates from the data stored within it. Furthermore, all of the fields b) to e) above are represented in a structured, standardized and machine-readable format. Namely, the plain English question or description of the query can be described via an *rdfs:comment,* the query itself via *sh:select, sh:ask, sh:update, sh:construct* or *sparql-examples:describe [18]* (according to the type of the SPARQL query, *i.e.* ASK, SELECT etc.). For multilingual support in the examples, all questions are annotated with a corresponding language tag (e.g. "@en" for English). The set of queries in this work only includes Ask, Select and Describe queries. As DESCRIBE queries are defined in SPARQL 1.1 and can not be used in SHACL, we introduce a new property and concept to represent this type of query, that is, *sparql-examples:describe* where the *sparql-examples:* prefix corresponds to *https://purl.expasy.org/sparql-examples/ontology#*. The target SPARQL endpoint(s) are described with *schema:target*. Note that there can be several such endpoints where the query is directly executable. The query reusability (without any modifications) stems from the adoption of the FAIR principles in the design of KGs across the entire SIB Semantic Web of Data, which makes them uniformly queryable when requesting the same type of information. For example, the query defined at [19] (see also SPARQL query shown in Listing 1) is usable as-is in at least three SPARQL endpoints, namely Bgee, OMA and UniProt. In the case of this example, it retrieves the list of species in each KG. The *schema:target* shows where users can run these queries and also demonstrates the benefit of sharing knowledge representation of common concepts and values. Finally, example queries can optionally be annotated with additional keywords (tags) using *schema:keywords* property.

To further illustrate how question-query pairs are structured, Listing 1 shows another example of a SPARQL query that retrieves all taxa in UniProt and its corresponding question in plain English. The SPARQL query is defined using the *sh:select* relation, while the corresponding

natural language question (i.e., the question in plain English that corresponds to the query) is always assigned to the *rdfs:comment* relation. In the case of a DESCRIBE query, the main change is replacing *sh:select* property with *spex:describe*, followed by the text of the query, i.e. "DESCRIBE <URL>". The type of the DESCRIBE example query will be only *sh:SPARQLExecutable*. A concrete case can be seen at [20].

```
PREFIX ex: <https://sparql.uniprot.org/.well-known/sparql-examples/>
PREFIX sh: <http://www.w3.org/ns/shacl#>
PREFIX rdf: <http://www.w3.org/1999/02/22-rdf-syntax-ns#>
PREFIX rdfs:<http://www.w3.org/2000/01/rdf-schema#>

ex:001 a sh:SPARQLSelectExecutable, sh:SPARQLExecutable ;
    sh:prefixes _:sparql_examples_prefixes ;
    rdfs:comment "Select all taxa from the UniProt taxonomy"@en ;
    sh:select """PREFIX up: <http://purl.uniprot.org/core/>
SELECT ?taxon
FROM <http://sparql.uniprot.org/taxonomy>
WHERE
{
    ?taxon a up:Taxon .
}""" ;
    schema:target <https://sparql.uniprot.org/sparql/> ;
    schema:keywords "taxa" .
```

Listing 1. Question-query pair to select all UniProt taxa. We define this as a *sh:SPARQLSelectExecutable* where the question is assigned to *rdfs:comment* (with an English language tag annotation), while its corresponding SPARQL query in *sh:select*. The *schema:target* relation is used to define the target SPARQL endpoint where the query is executable. Optionally, the *sh:prefixes* relation is also stated to report all prefixes needed to run the query. This example has been uploaded to the UniProt SPARQL endpoint and can be viewed and executed at [19] .

After describing the SPARQL examples, we also store them in a standardized location directly as a subgraph within the KG that they target - that is, as part of the same SPARQL endpoint as the data they act on - so as to enhance their findability. This allows both users and services to uniformly retrieve examples of interest, or to aggregate information about the collection of examples available in each resource, by directly querying the SPARQL endpoints themselves. For example, users can query about all examples that target a specific endpoint, or all examples - across endpoints - that are annotated with a keyword of interest, by running a dedicated select SPARQL query, for example filtering on the *schema:keywords* property or on the text of the question itself (see Listing 2). Our GitHub repository includes more such pointers in the section “Querying for queries”. In addition, the standardized representation enables reusability of services across KGs with minimal modifications and without reliance on a centralized repository. We also provide in our GitHub repository [13] a script to merge the examples targeting a given endpoint as a single *.ttl* file that can be uploaded to a dedicated subgraph (e.g., as a named graph) within each KG. Across SIB, we have chosen to upload examples to a named graph

composed of the SPARQL endpoint web address and the *".well-known/sparql-examples"* suffix. Examples can then be assigned a unique identifier, more precisely, an Internationalized Resource Identifier (IRI), which may also be resolvable, *i.e.* allow access to the query using standard HTTP(S) for resource maintainers that want to associate specific webpages to the queries. For example, the query that retrieves all taxa over the UniProt SPARQL endpoint can be accessed at [19].

Once uploaded in the SPARQL endpoint, the examples themselves can be processed (for example, to search for relevant examples on a given topic) through simple SPARQL queries. Listing 2 shows how to retrieve example question-query pairs where the question contains the keyword "*species*" from a given endpoint. The following query can be directly executed, for example, over the UniProt SPARQL endpoint [21] or the Bgee SPARQL endpoint [22]:

```
PREFIX rdfs: <http://www.w3.org/2000/01/rdf-schema#>
PREFIX sh: <http://www.w3.org/ns/shacl#>
SELECT * WHERE {
  ?ex sh:select ?query .
  ?ex rdfs:comment ?question.
  FILTER (contains(?question, "species")) }
```

Listing 2. Query to retrieve all examples that include questions containing the keyword "*species*" from a SPARQL endpoint where formatted SPARQL examples have been uploaded

Finally, the landing page of our open GitHub repo provides an extensive description of how to apply our methodology to other KGs (RDF data stores). We include instructions and automated scripts to help describe new examples (i.e., query-question pairs), as well as explaining where such examples should be stored, how they can be queried (*i.e.,* "querying for queries"), tested automatically etc. To help KG maintainers check whether their endpoint meets all criteria required for metadata annotation, we have also developed an automated checker available online [23]. The checker solely requires a SPARQL endpoint URL to validate the existence of properly formatted metadata, such as SPARQL examples and the Vocabulary of Interlinked Datasets (VoID) [24] description, in the given endpoint. VoID is an RDF Schema vocabulary for expressing metadata about RDF datasets. It may be used to quantitatively describe relations between different datasets, or their subsets. For example, a VoID description will list classes and predicates used in a dataset and their cardinalities, i.e. how often they occur. For endpoints which do not have the full metadata available, the checker suggests concrete steps that can be taken, for example, to add the corresponding VoID description to the endpoint by using our open-source VoID generator [25].

Applications

In this section, we provide several applications facilitated by the collection of example queries, such as:

- Automated query testing across SIB endpoints (helping maintain query examples).

- Automatically generating graph visualizations of queries.
- Uniform query search and editing with a reusable SPARQL editor.
- Searching for scientific questions in the Bio-Query template interface.

All the applications can be deployed across the entire collection of questions and queries, owing to the standardized representation of examples that we have designed. The applications can be reused over any question-query collections that follow the approach described in the Methodology Section. All of the applications support knowledge graph maintainers in providing users with better support for querying their endpoints, addressing the challenges we have identified in the Introduction. More specifically, automated query testing ensures that the examples provided by maintainers remain up-to-date as the underlying data evolves. Query search and editing ensures that users with basic familiarity with SPARQL can find a relevant existing query and adapt it to their needs. Graph visualizations of queries further support users with little SPARQL knowledge to understand the general data flow of a query, with Bio-Query ultimately enables users to uniformly search for queries across the entire catalog and to adapt existing templated queries to their own information needs. We describe these applications in more detail in the following sections.

Tooling to help maintain SPARQL query examples

To help teams maintain their own SPARQL query examples, we provide a set of tools in a separate GitHub repository [26]. This includes testing for continuous integration, visualizations to help understand the queries, and a set of automated fixes that can remove common errors. We provide details on each of these components in the following sections.

Automated testing

Each SPARQL query is tested for syntactical correctness using two different parsers: the first provided by the Apache Jena [27] project, and a second by the eclipse RDF4j [28] project. The metadata about the query is tested using a set of SHACL rules with the RDF4j implementation of SHACL. These rules check if the structure of the metadata around the query matches the expected format, avoiding errors in the contributed examples. For example, the presence of all mandatory fields (query ID, query type, comment, target) is checked. Furthermore, the rules check if the query matches the metadata—for example, that all queries declared to be federated queries actually contain a SERVICE clause in the query text.

Optionally, we can also execute all queries on their remote target endpoints. We chose to only check that the queries retrieve at least one result, by programmatically changing the queries. More specifically, we add a limit which restricts the queries to only retrieve one result (i.e., a LIMIT [29] clause with a value of 1). This avoids introducing non-essential load on the tested SPARQL servers, since some queries would result in long-running times or high computational load. Finally, all SPARQL endpoints used in federated queries are tested to be alive and responsive to SPARQL queries. These tests are written in Java using JUnit 5 Jupiter [30] as the

test framework. Tests that do not require remote SPARQL endpoints are deployed as GitHub actions that run on each push to the shared git repository, ensuring the validity of newly contributed queries. Tests that require network or that access the remote SPARQL endpoints are only run on demand by the query writers and can be used as regression tests when changes are made to the target repositories (i.e., to ensure that existing queries are still valid). Many of the query examples are relatively complex and would therefore add significant traffic to the remote endpoints if executed frequently. We thus only run these tests on demand.

Enabling query visualization

Complex SPARQL queries are difficult to follow for the majority of users. To assist in understanding the existing question-query examples, we automatically generate Markdown [31] files with visual graphs and descriptions for each example query. To visualize these, we use Mermaid [32], a diagramming and charting tool inspired on the Markdown text syntax. Namely, we automatically generate for each example query a corresponding visual graph in Mermaid. The generated markdown files can be displayed as GitHub pages [33] or as Web pages in general (i.e., rendered HTML [34]). These Web pages can be then accessible, for example, via the web address used as their IRI. To facilitate the findability and understanding of the examples, we make the full collection, including Mermaid visualizations for each query, available online in GitHub Pages [35]. Figure 3 shows a simple Mermaid-based graph corresponding to an example query that asks for disease related proteins located inside the cell. This query can be executed at the UniProt SPARQL endpoint.

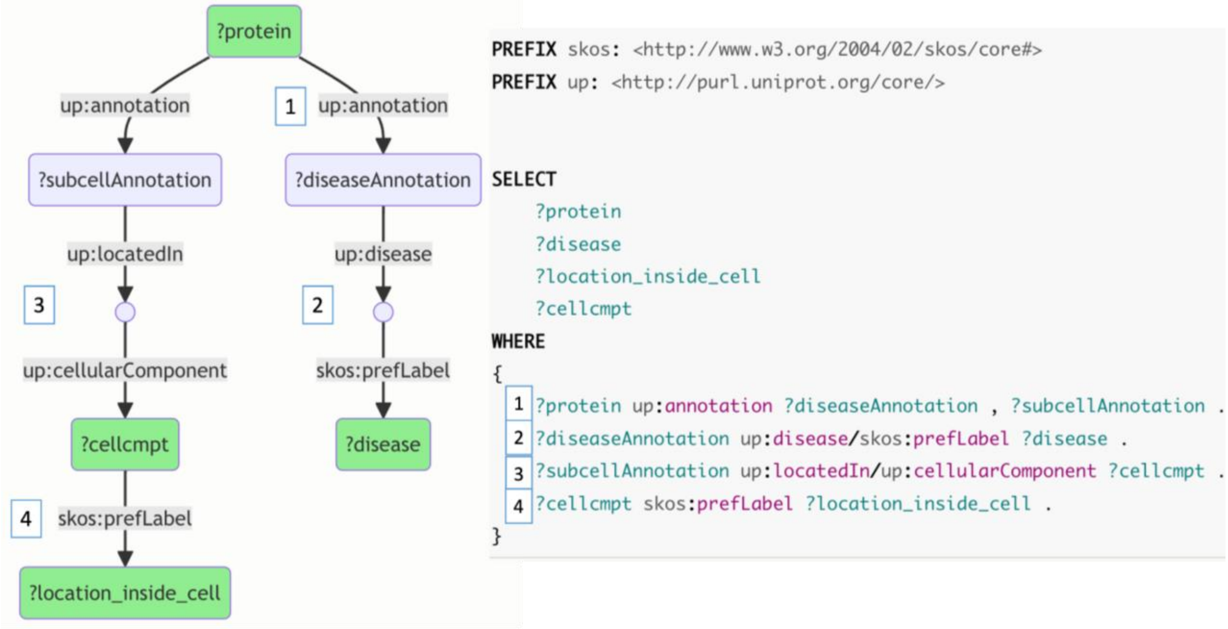

Figure 3. A graphical representation of [36] retrieving disease related proteins that are known to be located within the cell. This visualization is available in the GitHub pages of the collection of SPARQL examples [37]. In the figure, green cells show the projected variables, i.e. the variables that are selected to be included in the final result. The small circles act as intermediary, undefined variables (i.e., intermediary blank nodes) introduced by decomposing

property paths into individual triple patterns. The gray boxes shown as edge labels are the properties in the query triple patterns. In this figure, the numbered boxes shown on the left side of edges help to match the visualization to the corresponding triple patterns in the query shown on the right side.

Fixing query examples to be fully compliant with SPARQL 1.1

There are a number of query engines that have extended SPARQL 1.1 in non-standard ways. We implemented a module that attempts to “fix” these queries to be fully compliant with the SPARQL 1.1 specification. For example, Anzo and Blazegraph have implemented an extension for named subqueries. This named subquery extension is widely used in the Wikidata community. Nevertheless, these queries can often be semantically rewritten by following the SPARQL 1.1 specification. More precisely, our “fixer” approach parses the queries with the Blazegraph SPARQL parser and browses specific abstract syntax trees to look for such named subqueries and replace them with SPARQL 1.1 standard-compliant subqueries.

Another common mistake is that query examples might miss the prefix declaration required to make a query run. We store the common prefixes used in our resources, with an option for project specific prefixes, we can easily add missing ones to a query without further user interaction.

Query editing and searching

We extend the widely used Yasgui SPARQL environment [38] with a new section showing the queries in use. The code for this extension is available as a package at the *npm* software registry [39] and can be easily deployed by SPARQL endpoint maintainers using a single custom HTML element <sparql-editor/>. We welcome contributions to develop this further at [40]. The editor, available online [23], includes several useful features. It automatically pulls query examples from any SPARQL endpoint of interest (configurable in the top of the editor page) and presents them to the user alongside the editor, so users can easily reuse these queries to write their own by editing or extending them. It also provides a precise autocomplete based on the VoID descriptions that summarize an endpoint content. Whenever available, these descriptions are retrieved by the editor that queries the endpoint itself. The main information of interest processed by the editor from the VoID descriptions are the classes and properties that connect them. The VoID description is directly extracted from the SPARQL endpoint that is used by our extended Yasgui environment, therefore accurately reflecting the actual data. This ensures that only properties that are indeed stated, applicable according to the schema and the known type of a variable are suggested in the autocomplete dropdown menu. For further details on the VoID description generator used, see the corresponding GitHub repository [25].
In contrast, the authors of [41] propose a context-aware autocomplete approach that queries the SPARQL endpoint data directly. While effective for certain use cases, this method can face scalability challenges when dealing with large knowledge graphs not hosted in QLever triplestores. Our approach, which leverages precomputed metadata about the knowledge graph, ensures rapid responses across any triplestore, but requires endpoint maintainers to generate and maintain the VoID descriptions. These approaches are not mutually exclusive; we are

exploring the integration of QLever’s method into our SPARQL editor. This hybrid solution would enable the editor to dynamically select the most suitable autocomplete strategy for each endpoint, balancing scalability and responsiveness.

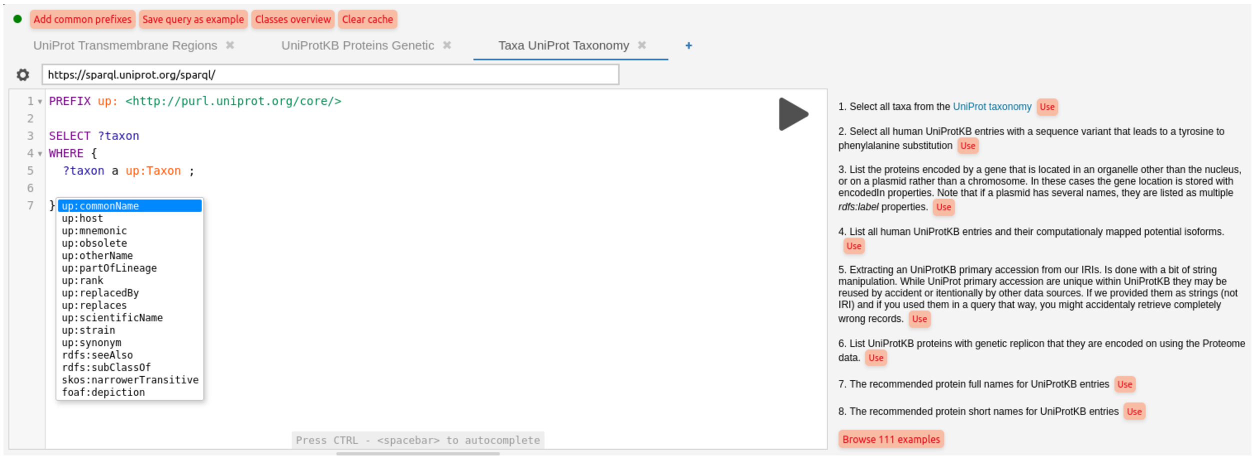

Figure 4. SPARQL query editor with context-aware autocomplete for the UniProt SPARQL endpoint. The list of query examples, classes and properties are automatically retrieved from the endpoint.

Integration in Bio-Query

The standardized representation of example queries facilitates their use in applications downstream. As an example, we have also incorporated the collection as-is in the Bio-Query [42] interface available online [43]. Figure 4 shows an example question and query from the collection. The questions are automatically assigned to categories named according to the resource that contributed them. The collection can be found under the “*SIB Example SPARQL queries*” category in the public Bio-Query interface.

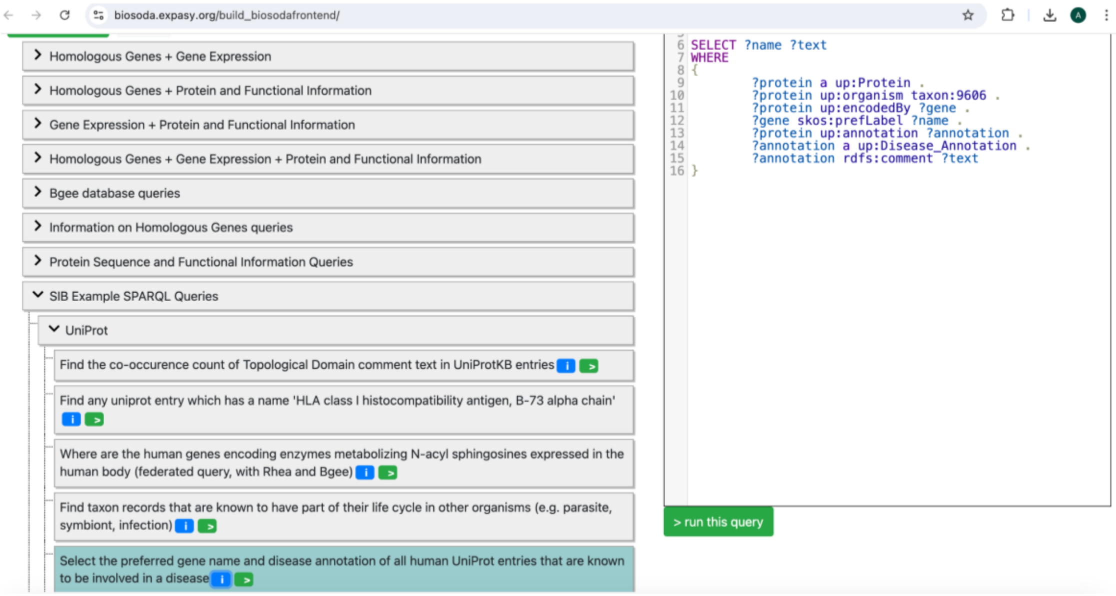

Figure 5. Example UniProt entry in the Bio-Query interface, integrated from the collection of SIB Example SPARQL queries. The collection is processed from the central GitHub repo in order to produce the JSON representation required by the interface. The standardization of the examples minimizes the effort to integrate these in the common interface, which can then act as a central hub to search for, and adapt, existing examples across SIB KGs.

Challenges and Lessons Learned

Efforts towards standardization are always faced with challenges at multiple levels. We have found that, even with community agreement on a common metadata format, simple details, such as non-uniform HTTP response headers expected across the different endpoints, can still hamper the uniform processing of the metadata. Moreover, even the queries themselves—although in principle portable across different triplestores—are not always reusable across different implementations. This is because, as mentioned previously, some query engines have extended SPARQL 1.1 in non-standard ways. An example is the way that “magic triples” are used to provide query execution hints [44] in Blazegraph or AWS Neptune. These “magic triples” need to be removed when moving to a different SPARQL endpoint, as they would otherwise refer to triples that do not exist, and would therefore break the original queries (i.e. their execution would give empty results).

At a higher-level, one of the biggest challenges we still face is that the collection of questions and queries were designed as generic guidelines for users, and not to be consumed by machines directly. This poses a set of unique challenges also in reusing them for the purpose of training and evaluating machine learning algorithms to automatically translate questions into equivalent SPARQL queries. End-user questions are often more abstract than their corresponding SPARQL queries, because the information searched by the user is not explicitly defined in the question. For example, let us consider the question: “What are the species available?”, in this question we do not know what is the exact information the user is looking for. Is the user searching for common names or scientific names of species, or both? Is the user

looking for a specific list of species identifiers of a given taxonomy? On the other hand, in a SPARQL query we often define the exact information to be retrieved (i.e., query projection). This impacts the retrieved results and potentially their amount. Therefore, while for an end-user the exact projected variables do not seem to be an essential point in the translation of a question to a query, for automatically evaluating an ML system, the set of projected variables must be precisely defined in the question (e.g., "*return only the species scientific names*", "*return only proteins and their associated gene names")* as opposed to being loosely defined, which would hamper a precise evaluation. Some questions in the catalog have also been designed in sequence, such that the text of one question is a follow-up to a previous one (e.g., "*Same as previous but with P0ABU7 as a query*"). These questions would need to be rephrased such that they are self-contained, given that most existing ML systems for translating natural language questions to queries treat each question as independent of the others.

Finally, an added challenge (common to all collections of question-query pairs) is that the SPARQL endpoints the queries target might change or not even be available anymore, rendering the queries harder to execute or reproduce. We acknowledge that ensuring long-term preservation of the corresponding data is a difficult task, however, precisely due to this reason we cannot maintain dedicated copies of the datasets targeted by the collection of queries. Instead, we rely on the resource providers to provide a long-term solution, which can be, for example, automatically redirect the existing SPARQL endpoint link to a site where the data is available at least in an archived format, from where users can deploy it locally.

Nevertheless, in spite of these challenges, the portfolio of reusable services that we have developed and deployed across independently managed SIB endpoints attests to the fact that even small steps towards standardization can have an important impact.

Related Work

Many existing works have proposed collections of Natural Language questions and corresponding SPARQL queries, usually designed as benchmarks to facilitate the task of automatically translating questions to queries or to evaluate federated query optimizers. However, existing collections suffer from shortcomings, which we mention below:

1) Non-federated: to the best of our knowledge, existing peer-reviewed published benchmarks focusing on translating questions to queries, do not consider federated queries and are usually centered around a single endpoint. We can mention here the examples of [45,46]. Even multi-dataset collections usually effectively address one single endpoint at a time, such as [45–47]. Consequently, existing KGQA systems also focus on simple, non-federated queries [48], often solely over DBPedia or Wikidata [49]. The existing collections of federated SPARQL queries, such as [50,51] are designed to merely evaluate SPARQL federation engines and do not include corresponding natural language questions.
2) Non-representative: crafting a large-scale benchmark manually is an extremely costly endeavor, therefore large collections (see [46]) have been generated semi-automatically, which does not ensure that the questions accurately represent the information needs of

real users. In contrast, our collection has been collected over time by different KG maintainers across the SIB, where most examples reflect questions asked by real users of the data.

3) Small scale: the higher-complexity, human-written question-query collections, such as the QALD series (Question Answering over Linked Data challenge updated each year, e.g. [52]), only include a small number of queries. Datasets which have been proposed for benchmarking the performance of federated queries specifically similarly only include a small number of queries. As an example, LargeRDFBench [53] includes only 40 (federated-only) queries of varying complexity. Similarly, FedBench [51] is a very good resource for benchmarking federated queries (including federated queries joining more target endpoints than our collection, i.e. up to 16 sources), however there are only 36 queries in total. In contrast, our dataset includes over 1,000 question-query pairs, all of which have been human-written and collected over time from the different KG maintainers.
4) HAMAP as SPARQL [54] is also a collection of more than 4,000 queries for a specific goal, annotating proteins. However, these are not aimed at educating users to the capabilities of a system. Nor are they annotated with a text for what their purpose is.
5) SPIN [55], SPARQL Inference Notation, is a W3C member submission that informed some of the design decisions of SHACL. SPIN provides for SPARQL templates and has logic for describing arguments to queries. However, its focus on constraints lead its modeling logic to ignore the concept of using these queries to mark up examples.
6) The Wikidata project has a media wiki template [56] for SPARQL queries. However, this does not standardize the metadata about the queries, nor does it provide the necessary utilities to help users maintain their queries.

A systematic overview and comparison of federated query benchmarks is provided in [57]. However, the overview focuses on the performance aspect of federated query processing, as opposed to natural language question-to-query translation.

Conclusion

In this paper, we introduce a large collection of example questions and their corresponding SPARQL queries collected over time across the catalog of SIB KGs and beyond. Moreover, we have proposed a methodology to standardize the representation of these examples, facilitating their uniform processing by both users and services. Finally, we have presented a comprehensive portfolio of services that leverage the standardization of metadata across the collection of examples, including services for the automated testing, editing and visualizing of example queries. All of these are available open-source and can easily be adapted by other KG maintainers for their own purposes, provided that maintainers adopt our recommended approach.

In the future, we plan to extend the current methodology to support defining templates in the example queries, in other words, fields that can be dynamically filled by querying a dedicated API (e.g., a SPARQL query). This would be a generalization of our current format, avoiding

hard-coding literals in the example queries. We also plan to deploy an LLM-based solution to automatically translate user questions in natural language to machine-readable SPARQL queries, leveraging the collection presented here.

We encourage the community to contribute to our proposed metadata specification, towards FAIRer Knowledge Graphs, which will pave the way for improved Semantic Web Tools. The advent of new technologies, such as Large Language Models, provide an opportunity for enabling a wider audience, including researchers and the public at large, to fully benefit from the wealth of interconnected data available in distributed KGs. We believe that a set of representative metadata standards for KGs, including the SPARQL examples metadata proposed here, represent an important and pragmatic step forward in this direction.

Competing interests

The authors declare that they have no competing interests.

Acknowledgements

We acknowledge contributions by all members of the SIB Semantic Web Focus Group, as well as funding from the Swiss State Secretariat for Education, Research and Innovation (SERI). This work was also supported by the CHIST-ERA grant CHIST-ERA-22-ORD-09 under the SNF grant 20CH21_217482; the Swiss Open Research Data Grants (CHORD) in Open Science I, a program coordinated by swissuniversities, grant id “Swiss DBGI-KM”; and by SERI via the HORIZON-INFRA-2024-EOSC-01-03 project grant number 101188078 — FIDELIS.

UniProt is supported by the Swiss Federal Government through SERI, and by the National Human Genome Research Institute (NHGRI), Office of Director [OD/DPCPSI/ODSS], National Institute of Allergy and Infectious Diseases (NIAID), National Institute on Aging (NIA), National Institute of General Medical Sciences (NIGMS), National Institute of Diabetes and Digestive and Kidney Diseases (NIDDK), National Eye Institute (NEI), National Cancer Institute (NCI), National Heart, Lung, and Blood Institute (NHLBI) of the National Institutes of Health under grant U24HG007822.

Authors' Contributions

Conceptualization: A.S, J.B., V.E., T.M.F. Software: A.S, J.B., V.E., T.M.F. Investigation: A.S, J.B., V.E., T.M.F. Supervision: A.S, T.M.F. Writing, original draft: A.S, J.B., V.E., T.M.F. Writing, review and editing: all authors. Funding acquisition: A.S, J.B., T.M.F, S.D., Al. B.

Data Availability

The catalog of examples is available via github [13]. A set of tools to convert, test and help maintain SPARQL query examples is available via github [26]. An archival copy of the example catalog is also available via the GigaScience database, GigaDB [58]. Associated codes are licensed under the MIT license and sparql queries under dual licenses MIT and CC-BY-4.0. All referenced datasets are publicly available, while the services leveraging the examples are all available open-source. The links to each of these are provided as references in the article text.

Abbreviations

DBGI - Digital Botanical Gardens Initiative
FAIR - Findable, Accessible, Interoperable, Reusable
HTTP - Hypertext Transfer Protocol
KG - Knowledge Graph
KGQA - Knowledge Graph Question Answering system
LLM - Large Language Model
QALD - Question Answering over Linked Data
RDF - Resource Description Framework
RDFS - RDF Schema
SHACL - Shapes Constraint Language
SIB - Swiss Institute of Bioinformatics
SPARQL - SPARQL Protocol and RDF Query Language
TTL - Terse RDF Triple Language
VoID - Vocabulary of Interlinked Datasets

References

1. UniProt Consortium. UniProt: the Universal Protein Knowledgebase in 2023. Nucleic Acids Res. 2023;51: D523–D531.

2. Sima A-C, de Farias TM. On the potential of artificial intelligence chatbots for data exploration of federated bioinformatics knowledge graphs. arXiv [cs.AI]. 2023. Available: http://arxiv.org/abs/2304.10427

3. Zahera HM, Moussallem D, Ngomo A-CN. UniQ-Gen: Unified Query Generation across Multiple Knowledge Graphs. Available: https://papers.dice-research.org/2024/EKAW_UniQ-Gen/public.pdf

4. Expasy overview of SIB Knowledge Graphs. [cited 20 Feb 2025]. Available: https://www.expasy.org/search/SPARQL

5. Vrandečić D, Krötzsch M. Wikidata: a free collaborative knowledgebase. Commun ACM. 2014;57: 78–85.

6. Galgonek J, Vondrášek J. IDSM ChemWebRDF: SPARQLing small-molecule datasets. J Cheminform. 2021;13: 38.

7. Zdrazil B, Felix E, Hunter F, Manners EJ, Blackshaw J, Corbett S, et al. The ChEMBL Database in 2023: a drug discovery platform spanning multiple bioactivity data types and time periods. Nucleic Acids Res. 2024;52: D1180–D1192.

8. The Digital Botanical Gardens Initiative (DBGI). [cited 20 Feb 2025]. Available: https://www.dbgi.org/

9. Statistics over collection of SPARQL queries. [cited 20 Feb 2025]. Available: https://sib-swiss.github.io/sparql-examples/examples/algebra-statistics

10. SIB Swiss Institute of Bioinformatics RDF Group Members. The SIB Swiss Institute of bioinformatics semantic web of data. Nucleic Acids Res. 2024;52: D44–D51.

11. W3C Shapes Constraint Language (SHACL) Vocabulary. 20 Jul 2017 [cited 20 Feb 2025]. Available: https://www.w3.org/ns/shacl.ttl

12. W3C Recommendation RDF Schema 1.1 (RDFS). 25 Feb 2014 [cited 20 Feb 2025]. Available: https://www.w3.org/TR/rdf-schema/

13. SPARQL examples GitHub repository. [cited 20 Feb 2025]. Available: https://github.com/sib-swiss/sparql-examples

14. W3C Recommendation RDF 1.1 Turtle (Terse RDF Triple Language). 25 Feb 2014 [cited 20 Feb 2025]. Available: https://www.w3.org/TR/turtle/

15. Bgee SPARQL examples. [cited 20 Feb 2025]. Available: https://github.com/sib-swiss/sparql-examples/tree/master/examples/Bgee

16. W3C Shapes Constraint Language (SHACL) recommendation. 20 Jul 2017 [cited 20 Feb 2025]. Available: https://www.w3.org/TR/shacl/

17. SHACL RDF Serializations. [cited 20 Feb 2025]. Available: https://www.w3.org/ns/shacl

18. SPARQLDescribeExecutable definition. Available: https://purl.expasy.org/sparql-examples/ontology#SPARQLDescribeExecutable

19. Get all taxa example SPARQL query. [cited 20 Feb 2025]. Available: https://sparql.uniprot.org/.well-known/sparql-examples/1

20. UniProt DESCRIBE example query. [cited 20 Feb 2025]. Available: https://github.com/sib-swiss/sparql-examples/blob/master/examples/UniProt/15_describe_an_EMBL_cds.ttl

21. UniProt SPARQL endpoint. [cited 20 Feb 2025]. Available: https://sparql.uniprot.org/

22. Bgee SPARQL endpoint. [cited 20 Feb 2025]. Available: https://www.bgee.org/sparql/

23. SPARQL editor metadata checker. [cited 20 Feb 2025]. Available: https://sib-swiss.github.io/sparql-editor/check

24. W3C VoID Describing Linked Datasets with the VoID Vocabulary. 3 Mar 2011 [cited 20 Feb

2025]. Available: https://www.w3.org/TR/void/

25. VoID Generator GitHub repository. [cited 20 Feb 2025]. Available: https://github.com/JervenBolleman/void-generator

26. SPARQL examples utils. [cited 20 Feb 2025]. Available: https://github.com/sib-swiss/sparql-examples-utils

27. Apache Jena project. [cited 20 Feb 2025]. Available: https://jena.apache.org/

28. RDF4J project. [cited 20 Feb 2025]. Available: https://rdf4j.org/

29. SPARQL Offset Limit description. [cited 20 Feb 2025]. Available: https://www.w3.org/TR/sparql11-query/#sparqlOffsetLimit

30. JUnit Online User Guide. [cited 20 Feb 2025]. Available: https://junit.org/junit5/docs/current/user-guide/

31. GitHub Flavored Markdown Spec. [cited 20 Feb 2025]. Available: https://github.github.com/gfm/

32. Mermaid Diagramming and charting tool. [cited 20 Feb 2025]. Available: https://mermaid.js.org/

33. GitHub Mermaid Markdown pages. [cited 20 Feb 2025]. Available: https://github.blog/developer-skills/github/include-diagrams-markdown-files-mermaid/

34. Mermaid Web page guide. [cited 20 Feb 2025]. Available: https://mermaid.js.org/config/usage.html

35. SPARQL examples collection GitHub pages. [cited 20 Feb 2025]. Available: https://sib-swiss.github.io/sparql-examples/

36. UniProt SPARQL example 21. [cited 20 Feb 2025]. Available: https://sparql.uniprot.org/.well-known/sparql-examples/21

37. Visualization of UniProt SPARQL example 21. [cited 20 Feb 2025]. Available: https://sib-swiss.github.io/sparql-examples/examples/UniProt/21_where_are_genetic_disease_related_proteins_in_a_cell.html

38. Rietveld L, Hoekstra R. YASGUI: Not Just Another SPARQL Client. Advanced Information Systems Engineering. Berlin, Heidelberg: Springer Berlin Heidelberg; 2013. pp. 78–86.

39. SPARQL endpoint NPM package. [cited 20 Feb 2025]. Available: https://www.npmjs.com/package/@sib-swiss/sparql-editor

40. SPARQL editor GitHub repository. [cited 20 Feb 2025]. Available: https://github.com/sib-swiss/sparql-editor

41. Bast H, Kalmbach J, Klumpp T, Kramer F, Schnelle N. Efficient and effective SPARQL autocompletion on very large knowledge graphs. Proceedings of the 31st ACM International Conference on Information & Knowledge Management. New York, NY, USA:

ACM; 2022. doi:10.1145/3511808.3557093

42. Sima AC, Mendes de Farias T, Zbinden E, Anisimova M, Gil M, Stockinger H, et al. Enabling semantic queries across federated bioinformatics databases. Database . 2019;2019. doi:10.1093/database/baz106

43. BioQuery template search interface. [cited 20 Feb 2025]. Available: https://biosoda.expasy.org/build_biosodafrontend/

44. SPARQL query execution hints. [cited 20 Feb 2025]. Available: https://docs.aws.amazon.com/neptune/latest/userguide/sparql-query-hints.html

45. Trivedi P, Maheshwari G, Dubey M, Lehmann J. LC-QuAD: A Corpus for Complex Question Answering over Knowledge Graphs. The Semantic Web – ISWC 2017. Springer International Publishing; 2017. pp. 210–218.

46. Dubey M, Banerjee D, Abdelkawi A, Lehmann J. LC-QuAD 2.0: A Large Dataset for Complex Question Answering over Wikidata and DBpedia. The Semantic Web – ISWC 2019. Springer International Publishing; 2019. pp. 69–78.

47. Kosten C, Cudré-Mauroux P, Stockinger K. Spider4SPARQL: A complex benchmark for evaluating knowledge graph question answering systems. 2023 IEEE International Conference on Big Data (BigData). IEEE; 2023. pp. 5272–5281.

48. Zafar H, Napolitano G, Lehmann J. Formal Query Generation for Question Answering over Knowledge Bases. The Semantic Web. Springer International Publishing; 2018. pp. 714–728.

49. Diefenbach D, Singh K, Maret P. WDAqua-core1: A Question Answering service for RDF Knowledge Bases. Companion Proceedings of the The Web Conference 2018. Republic and Canton of Geneva, CHE: International World Wide Web Conferences Steering Committee; 2018. pp. 1087–1091.

50. Aimonier-Davat J, Nédelec B, Dang M-H, Molli P, Skaf-Molli H. FedUP: Querying large-scale federations of SPARQL endpoints. Proceedings of the ACM Web Conference 2024. New York, NY, USA: ACM; 2024. pp. 2315–2324.

51. Schmidt M, Görlitz O, Haase P, Ladwig G, Schwarte A, Tran T. FedBench: A benchmark suite for federated semantic data query processing. The Semantic Web – ISWC 2011. Berlin, Heidelberg: Springer Berlin Heidelberg; 2011. pp. 585–600.

52. Usbeck R, Yan X, Perevalov A, Jiang L, Schulz J, Kraft A, et al. QALD-10 – The 10th challenge on question answering over linked data. Semantic Web. 2023; 1–15.

53. Saleem M, Hasnain A, Ngonga Ngomo A-C. LargeRDFBench: A billion triples benchmark for SPARQL endpoint federation. Web Semant. 2018;48: 85–125.

54. Bolleman J, de Castro E, Baratin D, Gehant S, Cuche BA, Auchincloss AH, et al. HAMAP as SPARQL rules-A portable annotation pipeline for genomes and proteomes. Gigascience. 2020;9. doi:10.1093/gigascience/giaa003

55. SPIN SPARQL Inferencing Notation W3C Member Submission. [cited 20 Feb 2025]. Available: https://spinrdf.org/

56. Wikipedia Media Template. [cited 20 Feb 2025]. Available: https://www.wikidata.org/wiki/Template:SPARQL

57. Troumpoukis A, Charalambidis A, Mouchakis G, Konstantopoulos S, Digles D, Siebes R, et al. A Benchmark Suite for Federated SPARQL Query Processing from Existing Workflows. 2017. Available: https://research.manchester.ac.uk/en/publications/a-benchmark-suite-for-federated-sparql-query-processing-from-exis

58. Bolleman JT, Emonet V, Altenhoff A, Bairoch A, Blatter M, Bridge A et al. Supporting data for "A large collection of bioinformatics question-query pairs over federated knowledge graphs: methodology and applications" GigaScience Database. 2025. https://doi.org/10.5524/102691
