# Supplementary material for: A large collection of bioinformatics question–query pairs over federated knowledge graphs: methodology and applications
Source: Gigascience. 2025 May 16;14:giaf045. doi: 10.1093/gigascience/giaf045 (PMC12083453; doi:10.1093/gigascience/giaf045)
Supplement: giaf045_Authors_Response_To_Reviewer_Comments_Original_Submission [file giaf045_authors_response_to_reviewer_comments_original_submission.pdf]

Author's Response To Reviewer Comments

Dear Editors and Reviewers,

We would like to thank the reviewers for all their insightful comments, improvements and suggestions and the editors for considering our article for a possible publication in GigaScience.

We have revised the paper in order to take all comments into account. We thus hope that this new version of our article complies with the remarks and answers the questions raised by the reviewers.

The following paragraphs list point-by-point the issues identified by the reviewers, followed by our corresponding answers. Finally, to facilitate the review process, the modified and added sentences are highlighted in red in the revised version of our article.

NOTE: due to journal formatting requirements, all links and footnotes in the original submission have been replaced, throughout the manuscript, with references.

Reviewer #1:
REVIEWER:Targeting the life science community, the authors introduce a very valuable resource, aimed at facilitating the sharing and reuse of data through public knowledge graphs. The paper is extensive and very interesting, with many technical details. Although it is well-illustrated, it is not always easy to follow and I have some concerns.
AUTHORS: Thank you for this comment. We hope the revised version addresses all concerns pointed out.

REVIEWER: The readability of the paper could be improved with a better introduction for some of the standards and technologies. For example, SHACL or VoID could be better described, and their use more clearly justified.
AUTHORS: We have added more clarifications on these in the “Implementation” section.

REVIEWER: Moreover, the connection between the applications and the objectives of the paper is not always very clear. How does each of the proposed applications, in conjunction with the proposed query catalog, contribute to the challenges identified in the introduction?
AUTHORS: Thank you for raising this point, we have provided more clarifications in the Application section.

REVIEWER: Finally, the thousand queries, which are the core of the contribution, are not precisely, and quantitatively described. How are they representative of the content of the knowledge graphs ? Which parts of the ontologies are covered by the queries (used properties, classes) ?
AUTHORS: We provide a script to automatically compute coverage in our GitHub repo https://github.com/sib-swiss/sparql-llm/blob/main/notebooks/compare_queries_examples_to_void.ipynb . The script helps to provide an overview of which classes and predicates are not covered by the examples. However, this metric is also sometimes tricky to automate - consider the case where types are not explicitly defined in the queries - in this case, it can be hard to automatically infer the class (i.e. the rdf:type) of variables from the query text. Therefore, the coverage numbers reported by the script must be considered only as approximate. To the best of our knowledge, coverage is rarely (if ever) reported in published literature even of official benchmarks for natural language to query translation (such as QALD or LC-QUAD). For additional metrics regarding the complexity of our queries please also see https://sib-swiss.github.io/sparql-examples/examples/algebra-statistics.html .

REVIEWER: How many results are returned by the queries ?
AUTHORS: We thank the reviewer for the question. The main reason why we decided to not provide the number of query results is that the results may change overtime according to new releases of the RDF datasets, for example, UniProt regularly updates its knowledge graph on average every 3 months. Moreover, some of the queries have limits to assure that an example runs quickly, including subqueries with limits that may change the result count on different executions of the same SPARQL query. Our main

focus is on the translation of natural language questions to their equivalent queries (and indeed we expect this to be more stable over time, as schema changes are more infrequent than content-level updates).

REVIEWER:Queries are proposed by the data providers, but are they similar or different from the actual served queries (SPARQL endpoint query logs) ?
AUTHORS: Some examples are inspired by query logs, however, we only have limited access to these logs in the first place, due to Swiss federal law similar to GDPR. Moreover, this is out of the scope of this work since it will require not only to analyse the individual logged queries compared to our example query set but also guess or predict the corresponding natural language translation by capturing the user intent when formulating a SPARQL federated query. In addition, we are dealing with highly specialized scientific questions that can require cross-domain expertise, not necessarily covered by the authors' competencies. We will ask all authors

REVIEWER: Is it possible to quantify typical SPARQL patterns (property paths, aggregates, etc.) ?
AUTHORS: Yes, we can count the usage of basic SPARQL features, and we now include a markdown report of such in our output when converting the examples to Markdown. This full report can be found at https://sib-swiss.github.io/sparql-examples/examples/algebra-statistics .

REVIEWER: Regarding the discussion, the limits of the approach are not described. Nothing is said regarding the evolution of the knowledge graphs, both in terms of schema and content, and the costs of maintaining the query catalog up-to-date.
AUTHORS: The costs of maintaining the catalog up-to-date are split among the SPARQL endpoint maintainers. In the future, we could add more tests (that some of our teams already do) to check for queries that fail (or that return empty results where this is not expected). However, currently each KG maintainer is responsible for keeping their queries up-to-date.

REVIEWER: Apart from that, the proposed resource and its sharing with the broader community is very timely and essential, given the recent advances and extremely active research on LLMs.
AUTHORS: Thank you very much for the positive response!

Introduction
REVIEWER: "High quality" : what do you mean by "high-quality ? This would be useful to evaluate the proposed query catalog.
AUTHORS: Our specific angle is on realistic, non-synthetic questions that reflect the information needs of real users. Although this is a harder quality to evaluate automatically, we believe that having queries written by endpoint maintainers to support their users should result in higher quality queries, rather than those (semi-)automatically generated for benchmarking purposes (e.g. LC-Quad 1 and LC-Quad 2).

REVIEWER: "Representative" : same question, which leads to "how the proposed questions are representative with respect to the 11 knowledge graphs ?"
AUTHORS: We refer the reviewer to our response for the previous question - since endpoint maintainers themselves provide the examples (and they are the most knowledgeable authority with respect to their own data, while also having a direct interest in covering user information needs with the provided examples), we believe this results in the representativeness of the queries.

REVIEWER: "The rapid development and uptake of these services" could you provide evidence for that ? Do user issue SPARQL queries ? Are SPARQL queries wrapped through web APIs ? Generated by algorithms ?
AUTHORS: Across SIB SPARQL endpoints, we have both services and users that issue queries, with varying degrees of uptake (ranging from thousands of unique queries per month in the case of UniProt to a few tens for smaller KGs). All SPARQL endpoints described in the paper provide a webpage which enables direct querying with federated SPARQL 1.1 support. We have also started preliminary work to automatically generate R, Java and Python APIs to enable programmatic access to the SPARQL endpoints (e.g., https://github.com/TRIPLE-CHIST-ERA/sparql-api-codegen), but since this work is not mature enough we did not include it in this article. Moreover, Web APIs can also be generated by using existing tools like "grlc" (see https://github.com/CLARIAH/grlc) as pointed out in our git repository https://github.com/sib-swiss/sparql-examples?tab=readme-ov-file#generate-rq-files .
We will ask all authors

REVIEWER: A clarification is needed about the 10 or 11 SIB KGs ? (Introduction VS Sources sections)
AUTHORS: Thank you for pointing out this mismatch - indeed the information in the Sources is the correct one (10 KGs are SIB's, 1 is the result of an external collaboration)

Sources
REVIEWER: "source of information for machine learning": it's a bit vague, what would be the targeted ML tasks ?
AUTHORS: Thank you for the observation. Indeed, some of the machine learning tasks that would be possible are in the realm of translating natural language questions to SPARQL across heterogeneous Knowledge Graphs. We have added a specific mention of this now in the text.

REVIEWER: "… complexity of the queries": I'm not convinced by counting the average of triple patterns as an estimate of the complexity. What about the use of Unions, Negation, Service clauses ? The number of used variables, the use of property paths, etc. ?
AUTHORS: For some more detailed statistics we have now made available a script that counts automatically e.g. the number of aggregations, Service clauses etc. across the catalog of queries. The output of this script is available at https://sib-swiss.github.io/sparql-examples/examples/algebra-statistics.

REVIEWER: "small KG": a KG can also be large if you consider the number of class instances … or properties … not only the size of the schema.
AUTHORS: Thank you for this point, we have rephrased to explicit mention the size of the schema.

REVIEWER: "average number of TP per endpoint": to be clarified, is it per endpoint ? Is it per query ?
AUTHORS: Many thanks for this observation, indeed it is the average number of triple patterns per query, for each endpoint. We have added this clarification in the text.

Methodology
REVIEWER: I could not find anything on the SHACL spec about SPARQLExecutable concepts. As it is presented in the paper, it is unclear if it's something new or part of the spec. If it is introduced by the authors, it should be better described and motivated.
AUTHORS: Note that although SHACL is documented as a technical report and a W3C recommendation in (https://www.w3.org/TR/shacl/), the vocabulary terms we use are only fully described in its RDF serialization, that is either Turtle or RDF/XML in (https://www.w3.org/ns/shacl). We added this explanation in the "Implementation" section for clarity, and updated the links/references in the paper to refer directly to the SHACL vocabulary in turtle.

REVIEWER: "aggregate information about the collection of examples": Why ? How ?
We have added an example in the text and a clarification regarding where more specific queries can be found in "Querying for queries" in the GitHub repo. More aggregations, such as COUNT all queries that target a specific endpoint, are possible via similar mechanisms.

REVIEWER: "We provide a script": where ?
AUTHORS: The script can be found in the GitHub repository, section "Compile all query files into one file to upload to your endpoint".

REVIEWER: Listing 2: is it only possible to do "keyword-search" ? If so, this should be part of the discussion of the paper
AUTHORS: The user can have a better local "indexing" of the examples, leveraging their uniform representation. For example, users could upload examples into a vector search engine locally. Listing 2 is just a simple illustration to show that example queries can themselves be queried as any other RDF data, including for search purposes.

REVIEWER: VoID (and SHACL) should be better introduced/described
AUTHORS: Indeed, we have added more details in the "Implementation" section.

Applications
REVIEWER: The paragraphs on "automated testing" and "fixing SPARQL 1.1 syntax" should be better aligned with the main objectives of the paper
AUTHORS: The two paragraphs illustrate how automated testing can help support endpoint maintainers to keep the catalog of example queries up-to-date, which is in line with providing a catalog of high-quality, up-to-date representative queries for endpoint users (which is the goal of the paper).

REVIEWER: "decomposing graph paths": why not speaking about SPARQL property paths ?
AUTHORS: Done.

REVIEWER: The visualization of the BGP seems to be very useful for non SPARQL experts, congratulations !
REVIEWER: Query editor: how does it differ from Qlever auto-completion ?
AUTHORS: The Qlever UI query editor is not triplestore agnostic as compared to our approach. Although the autocomplete method proposed in [1] by the Qlever team is described to be applicable to other triple stores, the code is not publicly available anymore. Moreover, as stated in [2], the Qlever UI query editor is “a user-friendly interface for the Qlever SPARQL engine”, hence, it is not triple-store agnostic.
We acknowledge that a combination of both (our approach and Qlever) could provide overall even better results to the users, while remaining triplestore agnostic, therefore indeed we could foresee a collaboration with Qlever on this in the future.
We have added a discussion on this in the corresponding “Query editing and searching” section of the paper.

[1] https://ad-publications.cs.uni-freiburg.de/CIKM_sparql_autocompletion_BKKKS_2022.pdf
[2] https://github.com/ad-freiburg/qlever-ui?tab=readme-ov-file

REVIEWER: Bio-query : it is not totally clear how this example application supports the paper. What, in the methodology, allows to better categorize the 1000 queries in BioQuery ?
AUTHORS: The integration in BioQuery is shown as an example of the benefits of uniform SPARQL question-query metadata representation with respect to the services that can consume these examples. Representing all examples uniformly across different endpoints has facilitated their direct integration (and searchability) in the BioQuery interface.
In the future, we plan to add specific metadata to enable queries to be “templated” in the collection of examples, such that they can also be made configurable in the BioQuery interface (e.g. allowing users to replace specific entities in the questions, such as species or gene names, making the questions more easily adaptable to the needs of the users without requiring SPARQL knowledge, e.g. replacing “human” or “TP53” in “What are the orthologs of the human TP53 gene in the rat?” with the appropriate species / gene name).

Discussion
REVIEWER: "… to triples that do not exist": we understand implicitly that the query will not return any result, but it should be made explicit
AUTHORS: We have added the explicit mention in the text.

REVIEWER: The paragraph on "follow-up" questions is unclear
AUTHORS: We have rephrased this for clarity to “These questions would need to be rephrased such that they are self-contained, as most existing ML systems for translating natural language questions to queries treat each question in the collection as independent of the others.”

Related works
REVIEWER: In terms of structure, it seems more natural to describe related works before the discussion.
AUTHORS: The placement of “Related Works” is oftentimes dependent on the common practice in the community or discipline - in our work, we have opted to leave this towards the end, favoring the introduction of our specific sources and methodology first.

REVIEWER: Some federated sparql query benchmarks should be better described in the related works such as at least FedBench, or LargeRDFBench ([dice-group/LargeRDFBench: LargeRDFBench: A Billion Triples Benchmark for SPARQL Query Federation](https://github.com/dice-group/LargeRDFBench?tab=readme-ov-file#benchmark-queries)), where the natural language question is given for instance "Return for all US

presidents their party membership and news pages about them."
AUTHORS: Both FedBench and LargeRDFBench are good references for benchmarking federated query performance, however for the purposes of natural language question to query translation they both might be small, as they each include only about 40 queries pairs (i.e. the “large” aspect in LargeRDFBench indicates the size of the composing sources, which aggregate to over 1B triples, not the size of the query collection). We have added a description on both LargeRDFBench and FedBench in the Related Work.

Reviewer #2:
REVIEWER: Overall, this paper presents valuable resources that can benefit not only the semantic web community but also a broader audience of KG users. However, I believe certain aspects of the paper's organization and clarity could be enhanced. Below, I leave some suggestions:
AUTHORS: The authors thank the reviewer for supporting our work. We hope this revised version addresses all reviewers’ concerns.

REVIEWER: In the "Sources" section, I missed a brief description of the 10 KGs to understand better the different domains covered in the queries. Additionally, detailed statistics on the 1000 queries, such as the distribution of queries per KG and the proportion of federated queries, seem to be missing.
AUTHORS: For a description of the domains of the SIB KGs, we refer the reader to the NAR paper [1]. We have added more detailed statistics on the number of federated queries and other query characteristics online at https://sib-swiss.github.io/sparql-examples/examples/algebra-statistics.

[1] "The SIB Swiss Institute of Bioinformatics Semantic Web of data." Nucleic Acids Research 52, no. D1 (2024): D44-D51, https://doi.org/10.1093/nar/gkad902 .

REVIEWER: In the "Implementation" section, alongside the example of a SELECT query, it would be beneficial to include an example of a DESCRIBE query since new properties and concepts have been introduced to represent this query type.
AUTHORS: We have added a link to a concrete example, as well as a description of the specific metadata required in such cases, in the Implementation section.

REVIEWER: I think the organization of the "Application" section and the following subsections could be improved. The initial list of possible applications does not align with the structure of the subsequent subsections, leading to some confusion.
AUTHORS: Thank you for raising this point, we have changed the order of the initial list in the Application section to conform with the subsequent sections.

REVIEWER: In the "Query editing and searching" section, adding a figure similar to the one provided in the "Integration in Bio-Query" section to illustrate the features would enhance clarity.
AUTHORS: We thank the reviewer for the suggestion, we have now integrated a screenshot in the section.

REVIEWER: In the "Related Work" section, including a summarizing table where existing collections are listed in rows and evaluated against characteristics such as scale, the inclusion of federated queries, representativeness, etc., would provide a clear comparison.
AUTHORS: We have provided a more systematic overview in the Related Work section, including a federated benchmark survey paper. However, our goal is not necessarily to survey and compare with existing benchmarks, but to provide a realistic use case and collection of questions and queries collected over time within 11 publicly available bioinformatics SPARQL endpoints.

Minor Comments:
REVIEWER: On page 5, the sentence: "For example, the query defined at https://www.bgee.org/sparql/.well-known/sparqlexamples/1 could directly reference Listings 1.
Indeed, we have now added this reference.

REVIEWER: In the caption of Figure 3, replace the word "here" with the actual URL to ensure the link is explicit and accessible.
AUTHORS: Thank you for the suggestion, indeed the formatting has now been revised to explicitly add all URLs as references (at the end of the paper).

Cookie Preference Center

We use cookies which are necessary to make our site work. We may also use additional cookies to analyze, improve and personalize our content and your digital experience. For more information, see our Cookie Policy.

You may choose not to allow some types of cookies. However, blocking some types may impact your experience of our site and the services we are able to offer. See the different category headings below to find out more or change your settings.

Allow all

Manage Consent Preferences

Strictly Necessary Cookies

Always active

These cookies are necessary for the website to function and cannot be switched off in our systems. They are usually only set in response to actions made by you which amount to a request for services, such as setting your privacy preferences, logging in or filling in forms. You can set your browser to block or alert you about these cookies, but some parts of the site will not then work. These cookies do not store any personally identifiable information.

Functional Cookies

These cookies enable the website to provide enhanced functionality and personalisation. They may be set by us or by third party providers whose services we have added to our pages. If you do not allow these cookies then some or all of these services may not function properly.

Performance Cookies

These cookies allow us to count visits and traffic sources so we can measure and improve the performance of our site. They help us to know which pages are the most and least popular and see how visitors move around the site.

Targeting Cookies

These cookies may be set through our site by our advertising partners. They may be used by those companies to build a profile of your interests and show you relevant adverts on other sites. If you do not allow these cookies, you will experience less targeted advertising.

Cookie List

Clear

Apply Cancel
Consent Leg.Interest

Confirm my choices

Powered by onetrust
