# Supplementary material for: A large collection of bioinformatics question–query pairs over federated knowledge graphs: methodology and applications
Source: Gigascience. 2025 May 16;14:giaf045. doi: 10.1093/gigascience/giaf045 (PMC12083453; doi:10.1093/gigascience/giaf045)
Supplement: giaf045_Reviewer_1_Report_Original_Submission — Alban Gaignard -- 11/7/2024 [file giaf045_reviewer_1_report_original_submission.pdf]

Reviewer Report

Title: A large collection of bioinformatics question-query pairs over federated knowledge graphs: methodology and applications

Version: Original Submission **Date:** 11/7/2024

Reviewer name: Alban Gaignard

Reviewer Comments to Author:

GENERAL COMMENTSThis article introduces a large collection of natural language life science questions and their translation as SPARQL queries. This query catalog is complemented by a set of tools that facilitate the maintenance, testing, catgoriztaion and visualization of these queries.The introduction describes the challenges associated with writing SPARQL queries, particularly in the case of federated queries that require understanding the specific schemas of various data sources. It also highlights the needs related to the development of large language models, which could assist researchers in writing these queries. The "sources" section briefly presents the 11 SPARQL endpoints and a metric on query complexity. The authors then propose the methodology for building the query catalog, using SHACL, along with a collection of applications dedicated to testing, editing, categorizing, and visualizing queries. Finally, the authors discuss their results, present the state of the art, and outline future work.Targeting the life science community, the authors introduce a very valuable resource, aimed at facilitatating the sharing and reuse of data through public knowledge graphs. The paper is extensive and very interesting, with many technical details. Although it is well-illustrated, it is not always easy to follow and I have some concerns.The readability of the paper could be improved with a better introduction for some of the standards and technologies. For example, SHACL or VoID could be better described, and their use more clearly justified.Moreover, the connection between the applications and the objectives of the paper is not always very clear. How does each of the proposed applications, in conjunction with the proposed query catalog, contribute to the challenges identified in the introduction?Finally, the thousand queries, which are the core of the contribution, are not precisely, and quantitatively described. How are they representative of the content of the knowledge graphs ? which parts of the ontologies are covered by the queries (used properties, classes) ? How many results are returned by the queries ? Queries are proposed by the data providers, but are they similar or different from the actual served queries (SPARQL endpoint query logs) ? Is it possible to quantify typical SPARQL patterns (property paths, aggregates, etc.) ?Regarding the discussion, the limits of the approach are not described. Nothing is said regarding the evolution of the knowlede graphs, both in terms of schema and content, and the costs of maintaining the query catalog up-to-date.Apart from that, the proposed resource and its sharing with the broader community is very timely and essential, given the recent advances and extremely active research on LLMs.#### DETAILED COMMENTS##### Introduction- "High quality" : what do you mean by "high-quality ? this would be useful to evaluate the proposed query catalog.- "Representative" : same question, which leads to "how the proposed qestions are representative with respect to the 11 knolwedge graphs ?"- "The rapid development and uptake of these services" could you provide evidences for that ? Do user issue SPARQL queries ? are SPARQL queries wrapped through web APIs ?

generated by algorithms ?- A clarification is needed about the 10 or 11 SIB KGs ? (Introduction VS Sources sections)##### Sources- "source of information for machine learning": it's a bit vague, what would be the targeted ML tasks ?- "… complexity of the queries":- I'm not convinced by counting the average of triple patterns as an estimate of the complexity. What about the use of Unions, Negation, Service clauses ? the number of used variables, the use of property paths, etc. ?- "small KG": a KG can also be large if you consider the number of class instances … or properties … not only the size of the schema.- "average number of TP per endpoint": to be clarified, is it per endpoint ? is it per query ?##### Methodology- I could not find anything on the SHACL spec about SPARQLExecutable concepts. As it is presented in the paper, it is unclear if it's something new or part of the spec. If it is introduced by the authors, it should be better described and motivated.- "aggregate information about the collection of examples": why ? how ?- "We provide a script": where ?- Listing 2: is it only possible to do "keyword-search" ? if so, this should be part of the discussion of the paper- VoID (and SHACL) should be better introduced/described##### Applications- The paragraphs on "automated testing" and "fixing SPARQL 1.1 syntax" should be better aligned with the main objectives of the paper- "decomposing graph paths": why not speaking about SPARQL property paths ?- The visualization of the BGP seems to be very useful for non SPARQL experts, congratulations !- Query editor: how does it differ from Qlever auto-completion ?- Bio-query : it is not totally clear how this example application supports the paper. What, in the methodolgy, allows to better categorize the 1000 queries in BioQuery ?##### Discussion- "… to triples that do not exist": we understand implicitly that the query will not return any result but it should be made explicit- The paragraph on "follow-up" questions is unclear##### Related works- In terms of structure, it seems more natural to describe related works before the discussion.- Some federated sparql query benchmarks should be better described in the related works such as at least FedBench, or LargeRDFBench ([dice-group/LargeRDFBench: LargeRDFBench: A Billion Triples Benchmark for SPARQL Query Federation](https://github.com/dice-group/LargeRDFBench?tab=readme-ov-file#benchmark-queries)), where the natural language question is given for instance "Return for all US presidents their party membership and news pages about them."

Level of Interest

Please indicate how interesting you found the manuscript: Choose an item.

Quality of Written English

Please indicate the quality of language in the manuscript: Choose an item.

Declaration of Competing Interests

Please complete a declaration of competing interests, considering the following questions:

- Have you in the past five years received reimbursements, fees, funding, or salary from an organisation that may in any way gain or lose financially from the publication of this manuscript, either now or in the future?

- Do you hold any stocks or shares in an organisation that may in any way gain or lose financially from the publication of this manuscript, either now or in the future?
- Do you hold or are you currently applying for any patents relating to the content of the manuscript?
- Have you received reimbursements, fees, funding, or salary from an organization that holds or has applied for patents relating to the content of the manuscript?
- Do you have any other financial competing interests?
- Do you have any non-financial competing interests in relation to this paper?

If you can answer no to all of the above, write 'I declare that I have no competing interests' below. If your reply is yes to any, please give details below.

I declare that I have no competing interests

I agree to the open peer review policy of the journal. I understand that my name will be included on my report to the authors and, if the manuscript is accepted for publication, my named report including any attachments I upload will be posted on the website along with the authors' responses. I agree for my report to be made available under an Open Access Creative Commons CC-BY license (http://creativecommons.org/licenses/by/4.0/). I understand that any comments which I do not wish to be included in my named report can be included as confidential comments to the editors, which will not be published.

Choose an item.

To further support our reviewers, we have joined with Publons, where you can gain additional credit to further highlight your hard work (see: https://publons.com/journal/530/gigascience). On publication of this paper, your review will be automatically added to Publons, you can then choose whether or not to claim your Publons credit. I understand this statement.

Yes Choose an item.
