# Supplementary material for: A large collection of bioinformatics question–query pairs over federated knowledge graphs: methodology and applications
Source: Gigascience. 2025 May 16;14:giaf045. doi: 10.1093/gigascience/giaf045 (PMC12083453; doi:10.1093/gigascience/giaf045)
Supplement: giaf045_Reviewer_2_Report_Original_Submission — Rita Sousa -- 12/4/2024 [file giaf045_reviewer_2_report_original_submission.pdf]

Reviewer Report

Title: A large collection of bioinformatics question-query pairs over federated knowledge graphs: methodology and applications

Version: Original Submission **Date:** 12/4/2024

Reviewer name: Rita Sousa

Reviewer Comments to Author:

This paper introduces a collection of human-written natural language questions reflecting real-world queries posed by data users, paired with their corresponding SPARQL queries, including several federated queries. The collection includes queries over 10 KGs from different SIB groups. Additionally, the paper provides a suite of tools for automated testing, query visualization, SPARQL 1.1 compliance checking, or query editing.Overall, this paper presents valuable resources that can benefit not only the semantic web community but also a broader audience of KG users. However, I believe certain aspects of the paper's organization and clarity could be enhanced. Below, I leave some suggestions:- In the "Sources" section, I missed a brief description of the 10 KGs to understand better the different domains covered in the queries. Additionally, detailed statistics on the 1000 queries, such as the distribution of queries per KG and the proportion of federated queries, seem to be missing.- In the "Implementation" section, alongside the example of a SELECT query, it would be beneficial to include an example of a DESCRIBE query since new properties and concepts have been introduced to represent this query type.- I think the organization of the "Application" section and the following subsections could be improved. The initial list of possible applications does not align with the structure of the subsequent subsections, leading to some confusion.- In the "Query editing and searching" section, adding a figure similar to the one provided in the "Integration in Bio-Query" section to illustrate the features would enhance clarity.- In the "Related Work" section, including a summarizing table where existing collections are listed in rows and evaluated against characteristics such as scale, the inclusion of federated queries, representativeness, etc., would provide a clear comparison.Minor Comments:- On page 5, the sentence: "For example, the query defined at https://www.bgee.org/sparql/.well-known/sparqlexamples/1 could directly reference Listings 1.- In the caption of Figure 3, replace the word "here" with the actual URL to ensure the link is explicit and accessible.

Level of Interest

Please indicate how interesting you found the manuscript: Choose an item.

Quality of Written English

Please indicate the quality of language in the manuscript: Choose an item.

Declaration of Competing Interests

Please complete a declaration of competing interests, considering the following questions:

- Have you in the past five years received reimbursements, fees, funding, or salary from an organisation that may in any way gain or lose financially from the publication of this manuscript, either now or in the future?
- Do you hold any stocks or shares in an organisation that may in any way gain or lose financially from the publication of this manuscript, either now or in the future?
- Do you hold or are you currently applying for any patents relating to the content of the manuscript?
- Have you received reimbursements, fees, funding, or salary from an organization that holds or has applied for patents relating to the content of the manuscript?
- Do you have any other financial competing interests?
- Do you have any non-financial competing interests in relation to this paper?

If you can answer no to all of the above, write 'I declare that I have no competing interests' below. If your reply is yes to any, please give details below.

I declare that I have no competing interests.

I agree to the open peer review policy of the journal. I understand that my name will be included on my report to the authors and, if the manuscript is accepted for publication, my named report including any attachments I upload will be posted on the website along with the authors' responses. I agree for my report to be made available under an Open Access Creative Commons CC-BY license (http://creativecommons.org/licenses/by/4.0/). I understand that any comments which I do not wish to be included in my named report can be included as confidential comments to the editors, which will not be published.

Choose an item.

To further support our reviewers, we have joined with Publons, where you can gain additional credit to further highlight your hard work (see: https://publons.com/journal/530/gigascience). On publication of this paper, your review will be automatically added to Publons, you can then choose whether or not to claim your Publons credit. I understand this statement.

Yes Choose an item.
